# Melatonin alleviates lung injury in H1N1-infected mice by mast cell inactivation and cytokine storm suppression

**DOI:** 10.1371/journal.ppat.1011406

**Published:** 2023-05-18

**Authors:** Caiyun Huo, Yuling Tang, Xinsen Li, Deping Han, Qingyue Gu, Ruijing Su, Yunjie Liu, Russel J. Reiter, Guoshi Liu, Yanxin Hu, Hanchun Yang

**Affiliations:** 1 Key Laboratory of Animal Epidemiology of Ministry of Agriculture and Rural Affairs, College of Veterinary Medicine, China Agricultural University, Beijing, China; 2 Beijing Key Laboratory for Prevention and Control of Infectious Diseases in Livestock and Poultry, Institute of Animal Husbandry and Veterinary Medicine, Beijing Academy of Agriculture and Forestry Sciences, Beijing, China; 3 National Engineering Laboratory for Animal Breeding, Key Laboratory of Animal Genetics and Breeding of the Ministry of Agriculture and Rural Affairs, Beijing Key Laboratory for Animal Genetic Improvement, College of Animal Science and Technology, China Agricultural University, Beijing, China; 4 Department of Cell Systems and Anatomy, UT Health San Antonio, Long School of Medicine, San Antonio, Texas, United States of America; Johns Hopkins Bloomberg School of Public Health, UNITED STATES

## Abstract

Influenza A virus (IAV) H1N1 infection is a constant threat to human health and it remains so due to the lack of an effective treatment. Since melatonin is a potent antioxidant and anti-inflammatory molecule with anti-viral action, in the present study we used melatonin to protect against H1N1 infection under *in vitro* and *in vivo* conditions. The death rate of the H1N1-infected mice was negatively associated with the nose and lung tissue local melatonin levels but not with serum melatonin concentrations. The H1N1-infected AANAT^-/-^ melatonin-deficient mice had a significantly higher death rate than that of the WT mice and melatonin administration significantly reduced the death rate. All evidence confirmed the protective effects of melatonin against H1N1 infection. Further study identified that the mast cells were the primary targets of melatonin action, i.e., melatonin suppresses the mast cell activation caused by H1N1 infection. The molecular mechanisms involved melatonin down-regulation of gene expression for the HIF-1 pathway and inhibition of proinflammatory cytokine release from mast cells; this resulted in a reduction in the migration and activation of the macrophages and neutrophils in the lung tissue. This pathway was mediated by melatonin receptor 2 (MT2) since the MT2 specific antagonist 4P-PDOT significantly blocked the effects of melatonin on mast cell activation. Via targeting mast cells, melatonin suppressed apoptosis of alveolar epithelial cells and the lung injury caused by H1N1 infection. The findings provide a novel mechanism to protect against the H1N1-induced pulmonary injury, which may better facilitate the progress of new strategies to fight H1N1 infection or other IAV viral infections.

## Author summary

IAV is one of the most common respiratory pathogens, leading to acute lung injury and a high morbidity rate. Melatonin has important regulatory roles in modulating immune responses during acute inflammatory conditions. It is of great significance to explore the protection effect of melatonin against the H1N1-induced pulmonary injury. Here, we found that melatonin had essential impacts on protection against IAV infection in mice. For the first time, we proved that melatonin had the capacity to suppress the activation of mast cells and the associated inflammatory response during H1N1 infection. The molecular mechanisms involved melatonin down-regulation of gene expression for the HIF-1 pathway mediated by MT2 and inhibition of proinflammatory cytokine release from mast cells, ultimately, leading to a reduction in the migration and activation of the macrophages and neutrophils in the lung tissue. Consequently, melatonin suppressed apoptosis of alveolar epithelial cells and the lung injury caused by H1N1 infection. Our findings highlight a novel mechanism to protect against the H1N1-induced pulmonary injury, and also provide insights into the new strategies to fight IAV infections.

## Introduction

Influenza A virus (IAV) is one of the most common respiratory pathogens that cause annual seasonal epidemics, leading to a high morbidity rate and massive economic loss. Based on the report of the World Health Organization (WHO), the epidemics of influenza caused 3 to 5 million severe cases annually and among them 290 000 to 650 000 patients die from respiratory diseases globally. Since 2009, the pandemic of (H1N1) 2009 (PDM/09 H1N1) influenza virus has spread from Mexico and North America to over 215 countries with a high human-to-human transmissibility [1]. To date, WHO has reported over 1.45 million PDM/09 H1N1 cases throughout the world.

Vaccination remains the most effective preventive measure against influenza virus infection. However, due to the continuous emergence of new variants of IAV with antigenic drifts or shifts, the efficiency of vaccination does not always satisfy the real requirement [2]. Also, while several antiviral drugs including zanamivir (Relenza) and oseltamivir (Tamiflu) have been developed to treat IAV infection the emergence of drug-resistant strains limits their clinical application [3,4]. Thus, developing more effective remedies for IAV infection is an urgent agenda for researchers. In this respect, based on its multiple actions, melatonin is one of the molecules we have seriously considered.

Melatonin is a derivative of the essential amino acid tryptophan. Previously, it was considered to be produced uniquely in the pineal gland and retina to regulate circadian rhythms responsible for photoperiod changes in the environments, therefore, to modulate the seasonal reproductive behaviors in some vertebrates [5–7]. Recent studies have found that melatonin is synthesized in mitochondria [8]. Virtually, almost all tissues and cells including gastrointestinal tract, bone marrow, lymphocytes, cochlear membrane, Harderian glands, skin, lungs and brain have the capacity to synthesize melatonin; for example, high levels of melatonin are present in the gastrointestinal tract [9–13]. The level of melatonin in the pineal gland and blood exhibits an obvious circadian rhythm with high levels at night (dark phase) and baseline levels during the day. The greatest concentration of melatonin usually occurs near the middle of the dark period and then drops to basal levels in the morning [14]. These circadian changes are modulated by the master circadian clock, the suprachiasmatic nucleus, which responds to the light:dark cycle as detected by the eyes. Melatonin synthesis from serotonin is under control of two enzymes, i.e., arylalkylamine N-acetyltransferase (AANAT) and acetylserotonin-O-methyltransferase (ASMT) with the former being rate limiting [15]. Once synthesized in the pineal gland, melatonin is directly released into the cerebrospinal fluid and blood from where it has access to all cells and it also diffuses into other bodily fluids including urine, follicular fluid, seminal fluid, amniotic fluid, and milk.

Melatonin has an uncommonly broad spectrum of functions including sleep promotion, blood pressure regulation, mitochondrial maintenance, immune modulation, as an antioxidant as well as exhibiting antiviral actions [16]. As to its immune modulatory effects, melatonin administration significantly increases natural killer cell viability and chemotactic movement of immune cells [17,18]. Especially under conditions of immunosuppression in the mice with depressed expression of interleukin-1 (IL-1) and IL-6, melatonin administration up-regulated the expression of these cytokines to improve the immune response [19]. In contrast, under the uncontrolled immune/inflammatory conditions, melatonin significantly down-regulated neutrophil infiltration and inflammatory responses to limit tissue damage due to acute lung injury or pancreatitis [20,21]. Melatonin also significantly reduced the neutrophil and macrophage infiltrations, as well as astrocyte activation in the ischemic brain of rats [22]. The adhesion to and infiltration between endothelial cells are major factors contributing to the immune cell migration and accumulation at sites of inflammation. Melatonin significantly down-regulated the strong adhesion induced by leukotrienes B4 in endothelial cells and deceased interaction between neutrophils and endothelial cells [23]. The actions of IL-1β, another cytokine that induces pro-inflammatory cell infiltration and vascular smooth muscle proliferation, are negated by melatonin to maintain the vascular barrier intact [24]. Collectively, these findings document that melatonin has important regulatory roles in modulating immune responses during acute inflammatory conditions.

Acute lung injury is one of the most important causes of death of individuals infected with IAV, severe acute respiratory syndrome (SARS) coronavirus, middle east respiratory syndrome (MERS) coronavirus or SARS-CoV-2 (COVID-19) coronavirus. These infections promote inflammatory cell infiltration, hemorrhage and edema in the lungs, especially when an exaggerated inflammatory response occurs; this causes severe injury to the alveolar epithelium and vascular endothelial cells [25–28]. During the progression of these infections, circulating neutrophils infiltrate into the alveolar septa and eventually into the alveolar sacs; at these sites they generate abundant inflammatory mediators including proteolytic enzymes and reactive oxygen species (ROS) and reactive nitrogen species (RNS) which cause acute lung injury [29]. In addition to the neutrophils, the infiltrated macrophages attracted by chemokines also release pro-inflammatory cytokines and apoptosis-associated molecules to further aggravate lung damage [30]. The intratracheal administration of melatonin, however, markedly depressed the neutrophil and macrophage infiltration into lungs in a lipopolysaccharide-induced acute lung injury animal model, thereby alleviating the pulmonary injury [31]. The findings suggest that melatonin may also protect against pulmonary injury induced by IAV infection.

In previous study, we observed that mast cells play important roles in the pathogenesis of acute lung injury during IAV infection; mast cells aggravate lung injury by directly releasing inflammatory cytokines and other mediators which induce apoptosis [32,33]. Interestingly, when C57BL/6 mice and B6.Cg-Kit (W-sh) mice, both strains of which suffer with mast cell deletion, were infected with influenza virus A/WSN/33, severe acute lung injury and associated pathological alterations were only observed in C57BL/6 mice, but not in the B6.Cg-Kit (W-sh) mice [34].

Since mast cells are widely distribute in the respiratory mucosa, where they can be dramatically activated and actively participate in the first-line immunological response to IAV infection [33,35–40], herein we tested whether intranasal melatonin administration would also alleviate the lung injury by modulating mast cells immune responses during an IAV infection in mice.

## Results

### Effects of endogenous melatonin on the susceptibility of H1N1 infection in CD1 mice

**Fig 1:**
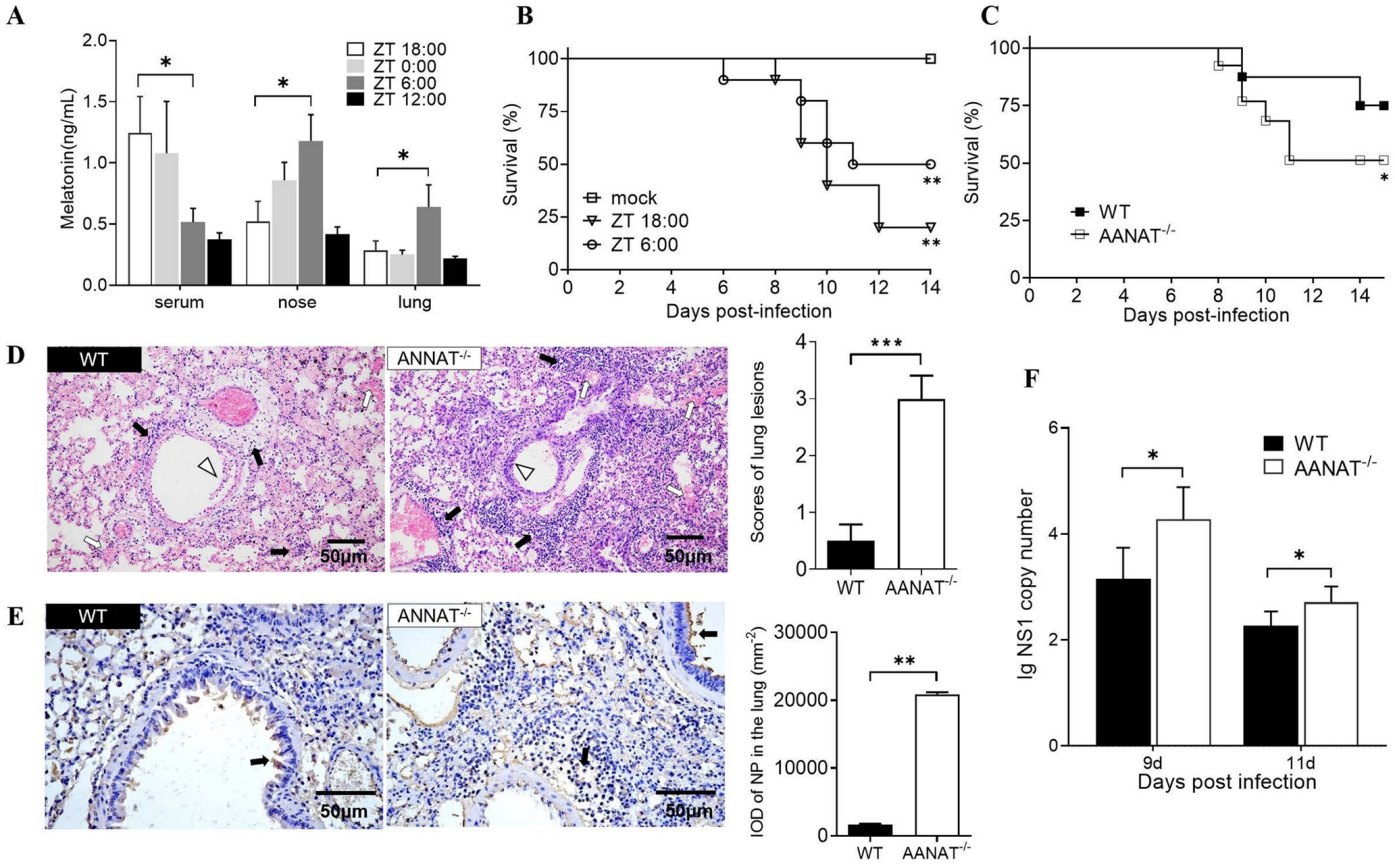
The blood and tissue melatonin circadian rhythms and their associations with the susceptibilities to the H1N1 infections in WT and AANAT^-/-^ CD1 mice. (A) Concentrations of melatonin in the serum, nose and lung of CD1 mice at different times of a day. Data are from three independent replicates. (B) Survival rates of CD1 mice after exposure to H1N1 virus at 0:00 AM and 12:00 noon, respectively (n = 10). (C) Survival rates of WT and AANAT^-/-^ CD1 mice after exposure to H1N1 virus, respectively (n = 7). (D) Lung pathology at day 9 of post-infection with H&E staining and scored by an examiner blinded to the study. Black arrows indicate lymphocyte infiltration. Hollow arrows indicate hemorrhage and hyperemia. Hollow triangles indicate desquamation of epithelial cells. (E) The expression of viral NP at day 9 of post-infection with IHC staining and scored by an examiner blinded to the study. Black arrows indicate positive signals. (F) The NS1 copy numbers in lung of mice at day 9 and 11 of post-infection with RT-qPCR. Values were means ± SEM. (*P < 0.05, **P < 0.01, ***P < 0.001) vs its respective group, determined by two-way ANOVA followed by Bonferroni statistical tests.

The levels of melatonin in the serum, nasal mucosa and lung tissue of CD1 mice were detected at different time points over a 24-hour period. The classic melatonin circadian rhythm with high level at night and low level during the day was observed in the serum (Fig 1A). Unexpectedly, in the nasal mucosa and lung tissue there was a reversed melatonin rhythm with the highest melatonin levels at noon (12:00; Zeitgeber Time 6:00) and low levels at midnight (0:00; Zeitgeber Time 18:00) (Fig 1A). Accordingly, the CD1 mice nasally inoculated with H1N1 virus at noon (12:00; Zeitgeber Time 6:00) had a significantly higher survival rate compared to the CD1 mice inoculated at midnight (0:00; Zeitgeber Time 18:00) (Fig 1B), which indicated that the elevated melatonin levels in respiratory system of CD1 mice impacted the susceptibility to the H1N1 infection.

To confirm the influence of melatonin on the susceptibility to H1N1 infection, the AANAT^-/-^ mice with aberrant melatonin synthesis were also infected with H1N1 virus. The results showed that the AANAT^-/-^ mice had a significantly higher lethal rate than that of their wide type counterparts (Fig 1C). When compared the histopathological alterations in the lung, nose and trachea at day 9 post-infection, the lungs of AANAT^-/-^ CD1 mice had more severe lesions than that of wide type including hyperemia, hemorrhage and desquamation of epithelial cells as well as increased infiltration of lymphocytes and inflammatory cells (Fig 1D and S1 Fig). The pathological scores of tissues were shown in Fig 1E. The IHC staining with an IAV NP antibody showed more positive cells in lungs of AANAT^-/-^ mice than that in the wide type, which were mainly distributed in the bronchiolar epithelial cells, alveolar epithelial cells and infiltrated immune cells. The IOD of IAV NP in the lungs showed the similar result as the pathological scores. Furthermore, the lung tissues at day 9 and 11 post-infection were collected for viral replication analysis by RT-qPCR. The results showed that NS1 copy numbers in lungs of AANAT^-/-^ mice were significantly higher than those in the wide type (*P* < 0.05) (Fig 1F), which was in accordance with the results of IHC staining. Taken together, the results indicate that the concentration of melatonin in local tissues is closely correlated with the pathogenicity in mice infected by H1N1 virus.

### Effects of exogenous melatonin administration on BALB/c mice infected with H1N1 virus

**Fig 2:**
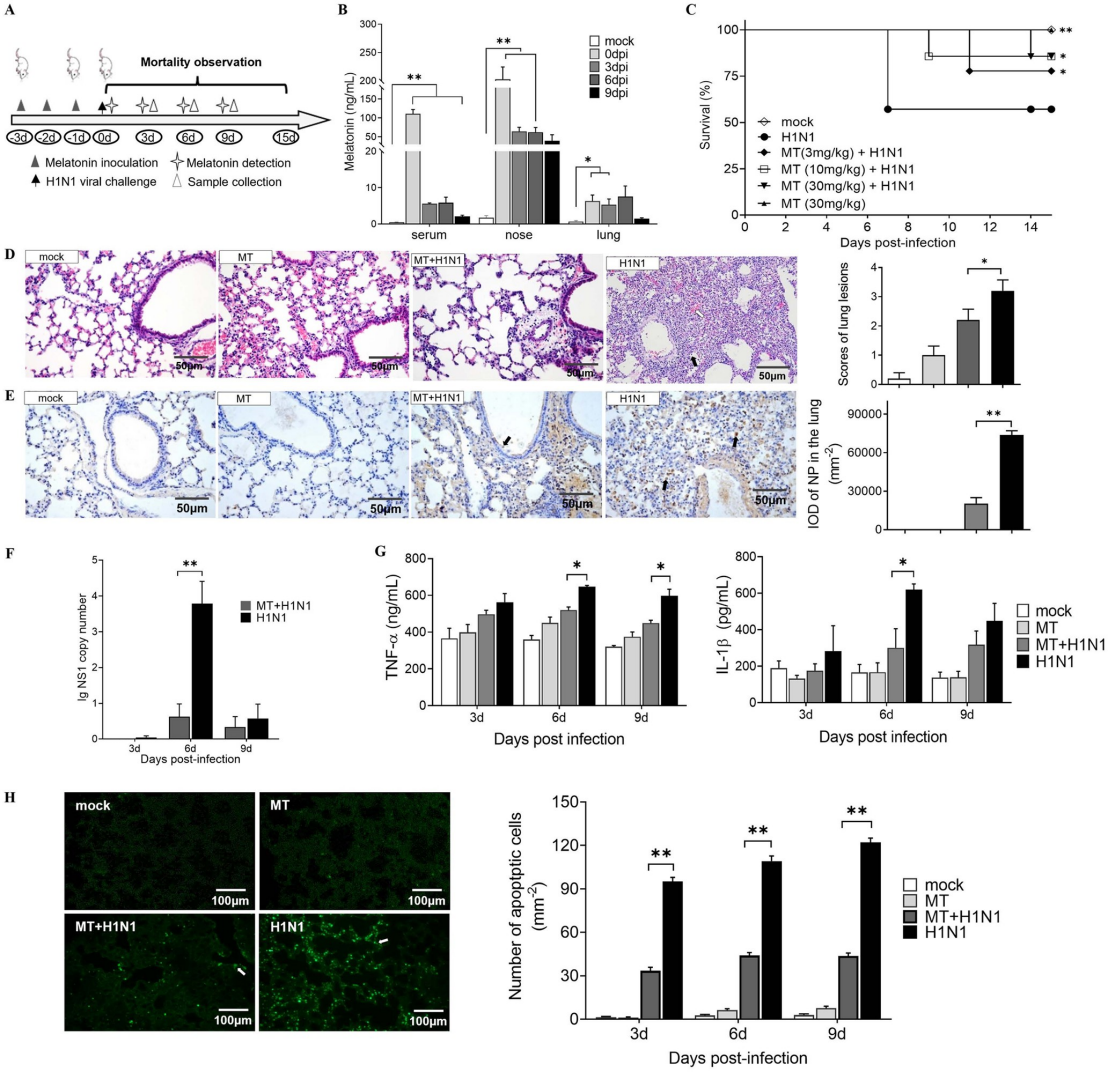
Effects of exogenous melatonin treatment on H1N1 infected BALB/c mice. (A) The schematic of experimental design with the time points of melatonin treatment (3, 10 and 30 mg/kg, respectively), H1N1 virus inoculation, melatonin detection and samples collection. (B) The levels of melatonin in the serum, nose and lung after intranasal melatonin administration detected by HPLC (n = 5). (C) Survival rates of H1N1-infected BALB/c mice treated with different doses of melatonin (n = 15). (D) Lung pathology at day 6 post-infection with H&E staining and scored by an examiner blinded to the study. Black arrows indicate lymphocyte infiltration. Hollow arrows indicate hemorrhage and hyperemia. (E) The expression of viral NP at day 6 of post-infection with IHC staining and scored by an examiner blinded to the study (n = 4). Black arrows indicate positive signals. (F) The NS1 copy numbers in lung of mice at day 3, 6 and 9 of post-infections, respectively with RT-qPCR (n = 5). (G) The expressions of TNF-α and IL-1β in lung of mice at day 3, 6 and 9 of post-infections, respectively, determined using ELISA (n = 3). (H) Apoptosis in lung of mice at day 3, 6 and 9 of post-infection, respectively, using the TUNEL assay. Green showed positive TUNEL signals. MT: melatonin. Values were means ± SEM. (*P < 0.05, **P < 0.01) vs its respective group, determined by two-way ANOVA followed by Bonferroni statistical tests.

To investigate whether exogenous melatonin administration provides protective effects against H1N1 infection, BALB/c mice which are congenital melatonin deficiency strain were given different doses of melatonin (3, 10 and 30 mg/kg, respectively) intranasally (Fig 2A). First, melatonin distribution in different tissues and different days after infection were detected. The results showed that the melatonin levels in all tissues tested were significantly increased after nasally-applied melatonin (Fig 2B). Interestingly, melatonin levels were significantly decreased in mice after H1N1 infection compared to prior to infection (Fig 2B). This indicated melatonin’s consumption due to virus infection; these findings are consistent with the results of a previous report [8]. The results also showed that all the three doses of melatonin significantly increased the survival rates of the mice compared to the infected control group (Fig 2C and S2 Fig). The 30 mg/kg melatonin caused severe body weight loss. Thus, the optimal dosage of melatonin which could improve the survival of H1N1-infected mice with fewer side effects was selected as 10 mg/kg for the rest of the studies.

The histopathological analysis showed that H1N1 virus infection caused desquamation of epithelial cells, hyperemia and hemorrhage as well as lymphocytes and inflammatory cell infiltration in the nose, trachea and particularly in lungs at day 6 post-infection (Fig 2D and S3 Fig). These pathological changes were significantly reduced with melatonin administration and the statistical analysis of pathological scores confirmed the results. With the aid of IHC staining, the H1N1 virus proteins were mainly located in the macrophages, alveolar epithelial cells, and bronchial epithelial cells in H1N1 infected mice (Fig 2E). Nevertheless, few positive signals were found in lung tissues of melatonin pre-treated mice compared to their infected counterparts and the same results were observed in the IOD of IAV NP (P < 0.01). RT-qPCR results also validated lower viral titers in melatonin pre-treated H1N1 infected mice than that in the infected control mice, especially at day 6 post-infection (*P* < 0.01) (Fig 2F). The mRNA levels of IL-1β and TNF-α in lungs were all significantly down-regulated by the melatonin administration at day 6 and/or day 9 post-infection compared to the infected control mice (*P* < 0.05) (Fig 2G).

Since apoptosis is associated with severe lung injury, the protective effect of melatonin on apoptosis in lungs of H1N1 infected mice also was assessed by TUNEL staining. The results showed that fewer apoptotic cells were identified in the lung tissues of melatonin pre-treated mice than that of untreated infected mice (Fig 2H).

### Effects of melatonin administration on mast cell activation in H1N1-infected mice

To identify the cell targeted by melatonin, pulmonary epithelial cells (A549 cells) were first selected for examination. The results showed that 10^−5^ mol/L melatonin treatment had no significant effects on H1N1 virus infected A549 cells relative to parameters of cell death, NP expression or apoptosis compared to the untreated control (S4A–S4C Fig). Based on our previous studies that mast cells aggravated apoptosis of the pulmonary epithelial cells by releasing the inflammatory mediators during an IAV infection [33], we focused on the potential effects of melatonin on mast cell activation.

**Fig 3:**
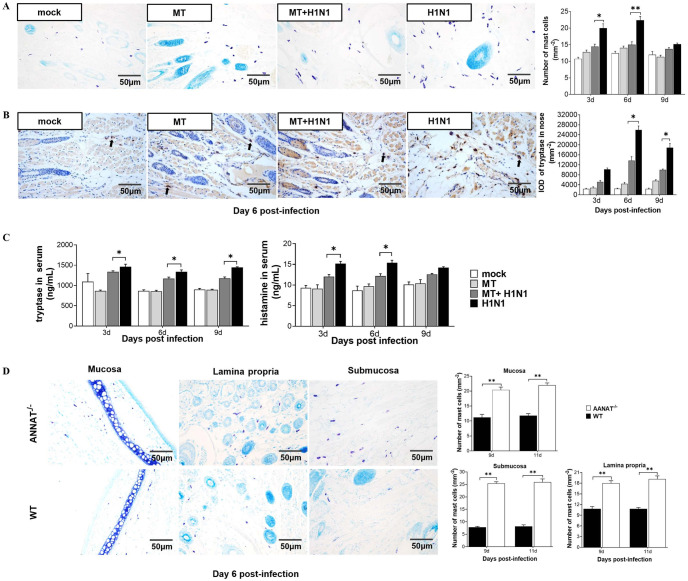
Effects of melatonin on mast cell activation and lung injury in H1N1 virus infected mice. (A-B) Number of mast cells and expression of tryptase in the nose with toluidine blue staining and IHC staining after melatonin (10 mg/kg) treatment and/or H1N1 virus inoculation (n = 4). (C) Expression of histamine and tryptase in the blood with ELISA (n = 3). (D) Number of mast cells in mucosa, lamina propria and submucosa of WT and AANAT^-/-^ CD1 mice, respectively, with toluidine blue staining. MT: melatonin. Values were means ± SEM. (*P < 0.05, **P < 0.01) vs its respective group, determined by two-way ANOVA followed by Bonferroni statistical tests.

By using toluidine blue staining to identify the mast cells, the results showed that the number of mast cells in the nasal mucosa and trachea in H1N1-infected BALB/c mice was significantly increased compared to the mock group while the intranasal administration of melatonin significantly reduced these elevated mast cell levels at day 3 and day 6 post-infection (Fig 3A and S5 Fig). In addition, the tryptase located in cytoplasm of mast cells in the nasal mucosa of H1N1-infected mice was significantly increased compared to the mock group with this rise being significantly down-regulated by melatonin administration (Fig 3B). The similar results were observed in the serum histamine and tryptase levels (Fig 3C). As expected, the mast cell numbers in the nasal mucosa of AANAT^-/-^ mice infected by H1N1 virus were significantly increased compared to their H1N1 virus infected WT (Fig 3D). The results further confirmed the mast cells activation was the target of melatonin’s action during H1N1 infection.

### Effects of melatonin on the proinflammatory mediators released by mast cells

**Fig 4:**
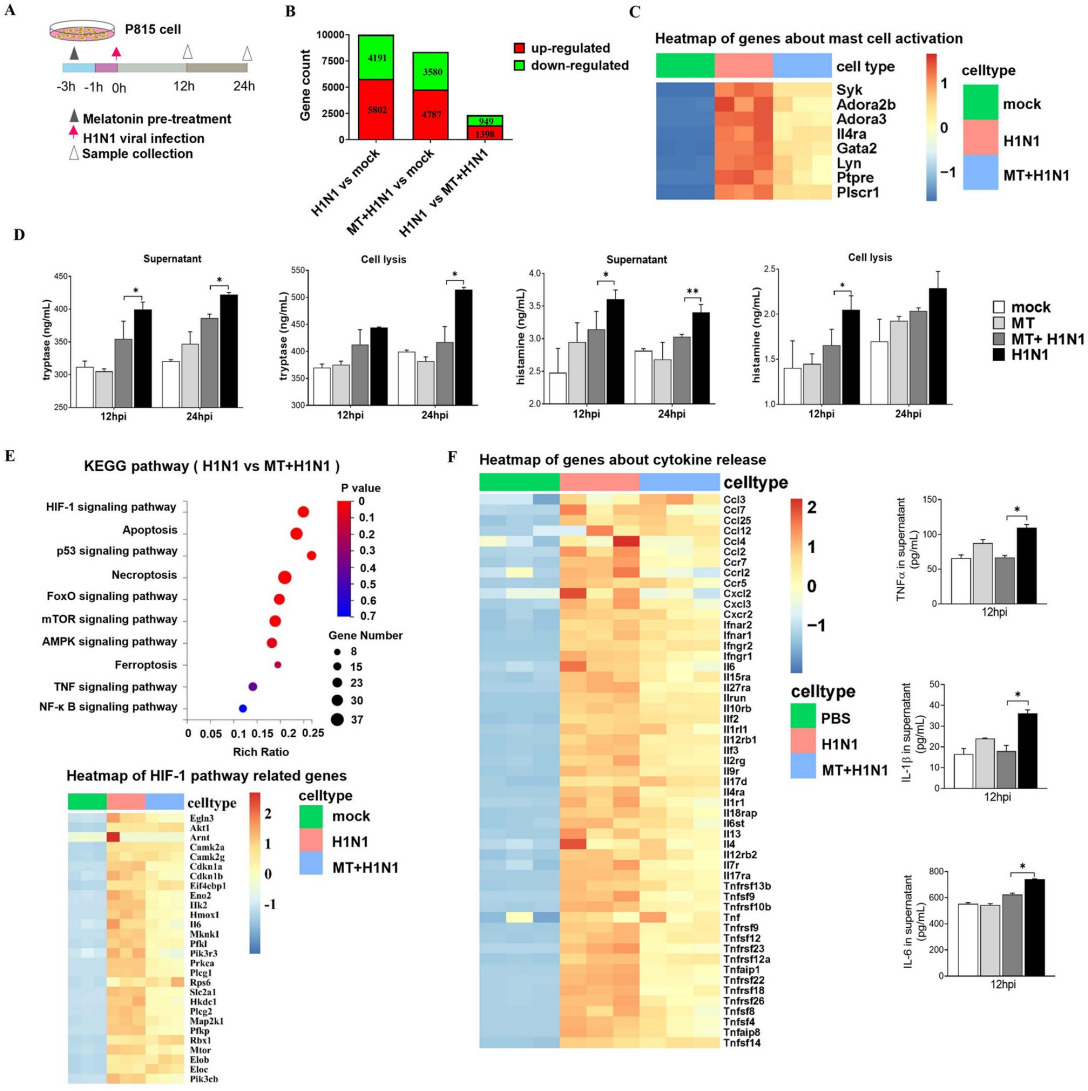
Effects of melatonin on the gene expressions in the mast cells. (A) The **s**chematic of experimental design with the time points of melatonin (10^−5^ mol/L) treatment, H1N1 virus infection, and sample collection from P815 mast cells. (B) The numbers of DEGs between different groups with RNA-seq. Venn diagrams showing the overlap of DEGs in each comparison group. (C) The genes related to mast cell activation among groups. MT: melatonin. (D) The levels of histamine and tryptase with ELISA (n = 3). (E) KEGG pathway enrichment analysis between groups, and the heatmap of genes about HIF-1 pathway enriched from the DEGs among groups. (F) The heatmap of genes about cytokine release enriched from the DEGs among groups, and the pro-inflammatory cytokines of TNF-α, IL-1β and IL-6 as measured using ELISA (n = 3). Values were means ± SEM. (*P < 0.05, **P < 0.01) vs its respective group, determined by two-way ANOVA followed by Bonferroni statistical tests.

To further understand the molecular mechanisms of melatonin on the mast cell activation, mouse mast cell line P815 was incubated with melatonin (Fig 4A) at the different concentrations (S6A Fig). Based on the results of cell viability estimated using the MTT assay, 10^−5^ mol/L melatonin was selected for remainder of the studies. The results showed that melatonin treatment significantly prevented mast cell damage caused by H1N1 virus infection compared to the infected control cells (S6B Fig). The transcriptomic profiles measured with RNA-seq analysis indicated that 5802 genes were up- and 4194 genes were down-regulated in the P815 cells after H1N1 infection compared to the mock. In the infected cells, melatonin treatment caused 4787 genes to be up- and 3580 to be down-regulated. Among them, 1398 up- and 949 down-regulated genes were shared by both the control and melatonin treated H1N1 infected P815 cells. (Fig 4B). Further analysis found that the mast cell activation related genes including *Syk*, *Adora2b*, *Adora3*, *Gata2*, etc. were significantly up-regulated in H1N1 infected P815 cells compared to the mock while this up-regulation was dramatically suppressed by melatonin treatment (Fig 4C). Similar results were observed regarding the levels of histamine and tryptase either in the supernatant or cell lysis buffer of H1NI-infected mast cells at 12 and 24 h of post-infection, i.e., melatonin treatment significantly reduced their levels compared to the untreated group (Fig 4D). KEGG pathway enrichment analysis of DEGs determined that 10 IAV-related pathways responded differently between melatonin treated and untreated H1N1-infected P815 cells. Among them, hypoxia-inducible factor-1 (HIF-1) pathway responded differently between the groups, i.e., the HIF-1 pathway related genes including Il6 and Pik3r3 were significantly down-regulated by melatonin treatment (Fig 4E).

**Fig 5:**
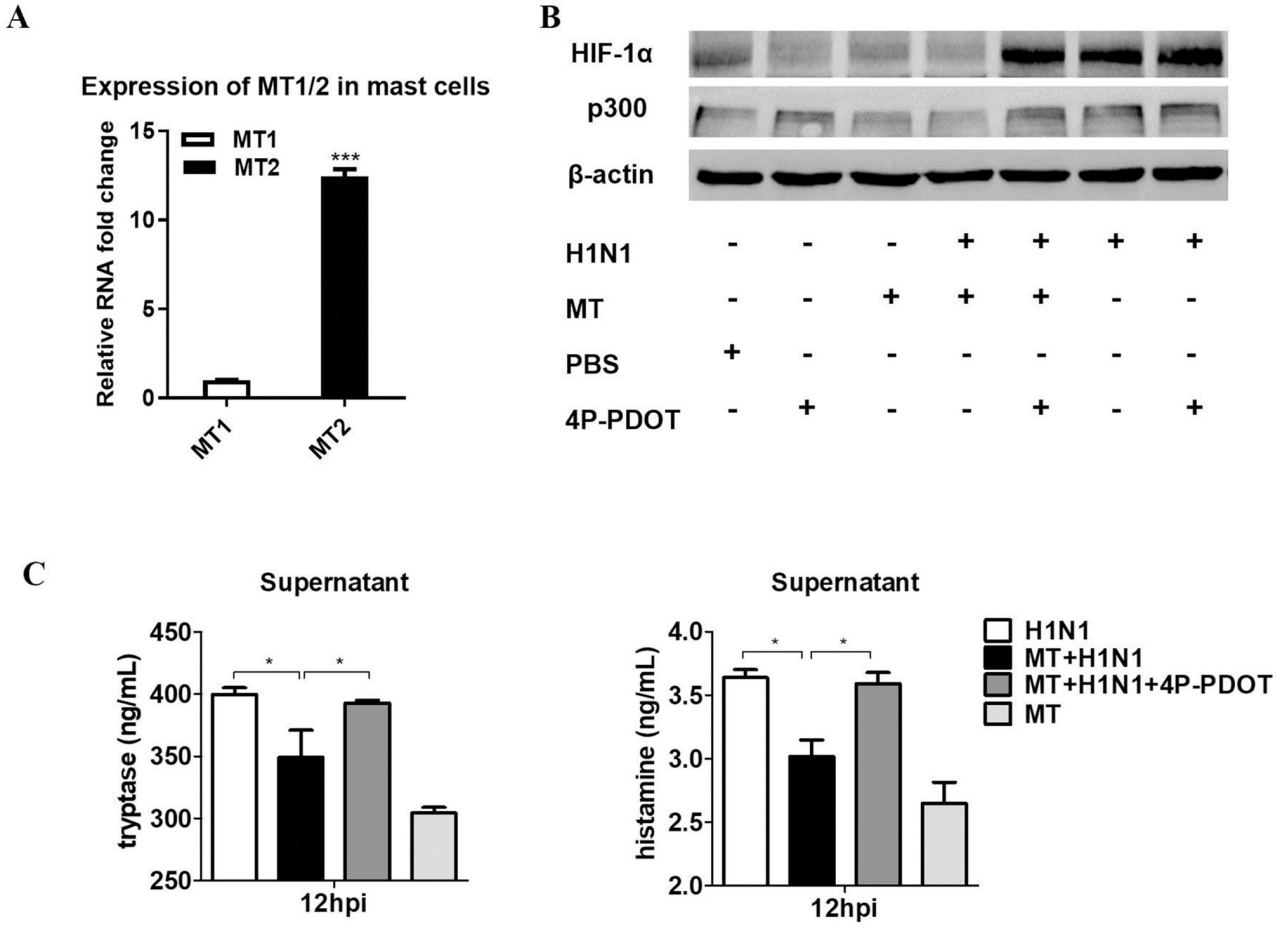
Effects of MT2 on mast cell activation. (A) The relative gene expression of MT1 and MT2 in P815 mast cells with RT-qPCR (n = 3). (B-C) The effects of melatonin (10^−5^ mol/L), and melatonin (10^−5^ mol/L) /4P-PDOT (10^−7^ M) on expression of HIF-1α and p300 measured by western blot, and the levels of histamine and tryptase measured by ELISA (n = 3) in mast cells. Values were means ± SEM. (*P < 0.05) vs its respective group, determined by two-way ANOVA followed by Bonferroni statistical tests.

To further validate the HIF-1 pathway, western blot was used to detect the protein expression of HIF-1α in mast cells in the two groups; we found that melatonin treated H1N1-infected P815 cells showed lower protein levels than those in the untreated H1N1-infected P815 cells, which was in accordance with above RNA-seq analysis (Fig 5B). The results indicated that mast cell inactivation by melatonin was probably mediated by inhibition of the HIF-1 pathway. As to the inflammatory response caused by the viral infection, DEGs analysis showed that the proinflammatory cytokine genes including *CCL7*, *IL-6*, *CXCL2*, *CCR4*, *CXCR2* and *TNF* in H1N1-infected P815 cells were significantly up-regulated while the expression of these up-regulated genes were markedly suppressed by melatonin treatment (Fig 4F). The absolute levels of TNF-α, IL-1β and IL-6 in P815 cells measured by ELISA confirmed the results from gene expression with RNA-seq analysis; thus, melatonin significantly reduced inflammatory cytokine levels at 12 h post-infection.

### Effects of melatonin membrane receptor 2 (MT2) on mast cell activation

Melatonin membrane receptors play important roles in mast cell physiology [41] with both the MT1 and MT2 having been identified on mast cell membranes [42]. In the current study we also confirmed the presence of MT1 and MT2 in P815 cells, but the expression of MT2 was much stronger than that of MT1 (*P* < 0.001) (Fig 5A). Therefore, MT2 was selected for receptor blocking study with the MT2 specific antagonist (4P-PDOT). The results showed that 10^−7^ M 4P-PDOT treatment almost completely blocked the effects of melatonin (10^−5^ mol/L) on mast cell activation. After 4P-PDOT treatment, melatonin had no significant effects on the elevated protein expression of HIF-1α, p300, levels of histamine and tryptase in H1N1-infected P815 cells; without MT2 receptor blockade, these parameters were suppressed by melatonin (Fig 5B and 5C).

### Effects of melatonin on alveolar epithelial cells apoptosis caused by mast cell activation

**Fig 6:**
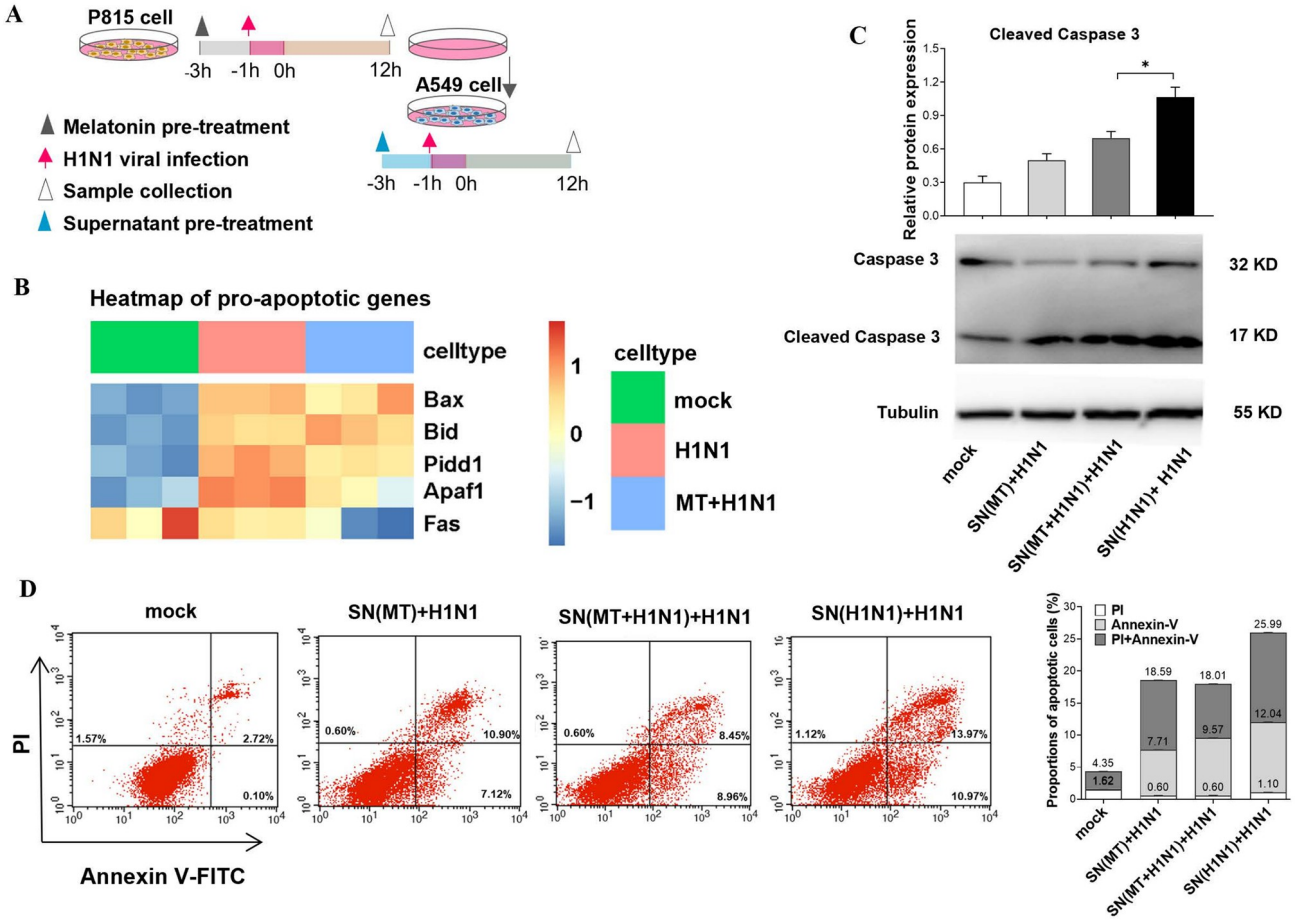
Effects of the supernatant from the melatonin treated P815 mast cells on A549 alveolar epithelial cells. (A) The schematic of experimental design with the time points—melatonin treatment (10^−5^ mol/L), H1N1 virus infection, and samples collection from P815 mast cells and A549 alveolar epithelial cells. (B) The heatmap of pro-apoptotic genes enriched from the DEGs among groups. (C-D) Apoptosis and expression of caspase 3 in A549 cells at 12 h after post-infection with flow cytometric analysis and western blot, respectively (four independent replicates). MT: melatonin. Values were means ± SEM. (*P < 0.05) vs its respective group, determined by two-way ANOVA followed by Bonferroni statistical tests.

To investigate the potential associations among mast cell activation, alveolar epithelial cell apoptosis and the effects of melatonin, the supernatants from H1N1 infected P815 cells with or without melatonin (10^−5^ mol/L) treatment were collected; these supernatants were then added to the culture medium of A549 cells (Fig 6A). The results showed that A549 cells incubated with supernatant from virus infected mast cells significantly up-regulated their pro-apoptotic genes of *Apaf1* and *Fas* compared to the PBS treated A549 cells while the A549 cells incubated with supernatant from virus infected mast cells with melatonin treatment significantly down-regulating these pro-apoptotic genes (Fig 6B). In addition, the protein expression of the apoptotic protein caspase3 was also evaluated. The level of cleaved caspase3 exhibited similar alterations as the *Apaf1* and *Fas* did in A549 cells (Fig 6C). The flow cytometric analysis further confirmed the results that melatonin reduced apoptosis frequency of alveolar epithelial cells by down-regulating the inflammatory response of mast cell activation caused by H1N1 infection (Fig 6D).

## Discussion

The development of more effective medications to treat IAV infection is an urgent agenda for medical scientists. In the current study, the potentially protective effects of melatonin on IAV infection as well as its molecular mechanisms have been systemically investigated. It is well known that blood melatonin level exhibits a circadian rhythm with its highest level at night and baseline levels during the day and a major portion of blood melatonin is released from the pineal gland in vertebrates. An aberrant blood melatonin circadian rhythm has been associated with a variety of disorders [43–45]. The circadian production and release of pineal melatonin is controlled by light/dark cycle as detected by the eyes. For example, mice are exposed to short-day or long-day conditions exhibit obviously-different melatonin cycles [46]. In the current study, when the CD1 mice were exposed to the natural light/dark cycle, the classic melatonin circadian rhythm was detected in their blood. In contrast, we found that the melatonin levels in the nose and lungs exhibited a reversed rhythmicity compared to that in the blood, i.e., their highest concentrations occurred at noon (12:00). This is an unusual observation but not surprising since perhaps all cells synthesize melatonin for its local protective actions [8]. One example is the gut which generates much more melatonin than the pineal gland; this melatonin is subsequently used locally to protect the normal functions of the gastrointestinal system including maintenance of gut microbiota [47]. This is consistent with current observations wherein cellular levels of melatonin in the tissues of the respiratory system including lungs rather than blood melatonin levels determined its protective actions against H1N1 virus infection. When the H1N1 virus was inoculated into the nasal cavity at noon (12:00 h), coincident with highest local melatonin levels in the nose and lung tissues, the death rate of the mice was significantly lower than that in mice inoculated in the middle of the night (0:00 h) at the time of the lowest melatonin levels in the respiratory tissues but the highest blood melatonin concentrations.

The studies using AANAT knockout mice confirmed the protective effects of melatonin. The AANAT^-/-^ mice exhibited aberrant melatonin synthetic function with low local melatonin production; when these mice were inoculated with the H1N1 virus they had a significantly higher death rate than that of the WT mice exposed to the virus. To further prove the protective effects of melatonin against an H1N1 infection, the natural melatonin deficiency mice, i.e., BALB/c mice, were selected for melatonin treatment. This strain has different genetic background with CD1 AANAT-/- mice but both with AANAT dysfunction. This selection would provide additional information as to whether melatonin exhibited antiviral action against different genetic background and this simulated to the clinical situation of human populations. As expected, this treatment dramatically increased the melatonin levels in blood, olfactory mucosa and lungs and, accordingly, significantly reduced the death rate of the BALB/c mice infected with H1N1 virus compared to their untreated infected controls. The results resemble the observations of Zhang et al. who found that intratracheal administration of melatonin markedly reduced the lipopolysaccharide-induced pulmonary injury of C57BL/6 mice [31].

To explore the potentially protective mechanisms of melatonin on H1N1 infection, we targeted the alveolar epithelial cells and the mast cells. The results showed that melatonin did not directly act on the alveolar epithelial cells; therefore, the subsequent studies focused on the mast cells. Mast cells are important innate immune cells and are widely distributed in the respiratory tract. They play important roles in immune responses by secreting inflammatory cytokines including TNF, IL-6, IL-1, and IFN-γ. These cytokines then up-regulate chemokine expression and release to induce the migration and activation of other immune cells which contribute to the inflammatory response [48]. Activated mast cells have been shown to be involved in the process of acute lung injury during influenza virus infection [33]. Mast cell activation after HIN1 infection was also observed in the current in vitro and in vivo studies and, importantly, the endogenously generated and exogenously administrated melatonin significantly suppressed mast cell activation and resultant lung injury. KEGG pathway enrichment analysis showed that the HIN1 infection induced mast cell activation involved the up-regulation of HIF-1 pathway. HIF-1 is the main regulator of cellular response to hypoxia and metabolic processes. It also impacts the degranulation and expression of inflammatory factors in mast cells [49]. As a consequence, dysregulation of the HIF-1 pathway is known to be associated with various diseases including breast cancer, *Mycobacterium tuberculosis* infection and severe acute respiratory syndrome caused by SARS-CoV-2 infection [50–52].

We had previously reported that H1N1 infection-associated mast cell activation was also mediated by HIF-1 signaling pathway [53]. In the current study, we further confirmed the previous observations and demonstrated that the inhibition of the HIF-1 pathway by melatonin in mast cell is mediated by MT2 membrane receptor since application of the MT2 specific antagonist, 4P-PDOT, blocked mast cell activation induced by the H1N1 virus. As already mentioned, activated mast cells release a variety of pro-inflammatory cytokines which exaggerated the immune response inducing acute lung injury and causing a high mortality [54–57]. Primary targets of the released cytokines are wandering macrophages and neutrophils which causes them to migrate to the respiratory tree; their migration into the lung tissue leads to elevations in apoptotic related factors, ROS and additional large quantities of proinflammatory cytokines, conventionally known as the cytokine storm [58,59]. The cytokine storm exacerbates damage of the alveolar epithelial and vascular endothelial cells which eventually culminates in severe edema, hemorrhage, additional neutrophil infiltration, and in alveolar collapse finally resulting in acute lung injury ^51^. Here, we observed that melatonin markedly reduced the pro-inflammatory cytokine secretion and down regulated chemokine gene expressions in mast cells. We thus hypothesize that mast cell inhibition is probably a major mechanism by which melatonin inhibits the inflammatory response during H1N1 infection.

In cultured cells, we found that melatonin did not directly protect the alveolar epithelial cells from apoptosis caused by H1N1 infection, but it did reduce the apoptosis of alveolar epithelial cells by modulating pro-apoptotic gene expression and inflammatory cytokine secretion by mast cells. The results provide additional evidence to support our speculation that the primary target of melatonin to protect against the lung injury caused by H1N1 infection is the mast cells, particularly to inhibition of mast cell activation.

**Fig 7:**
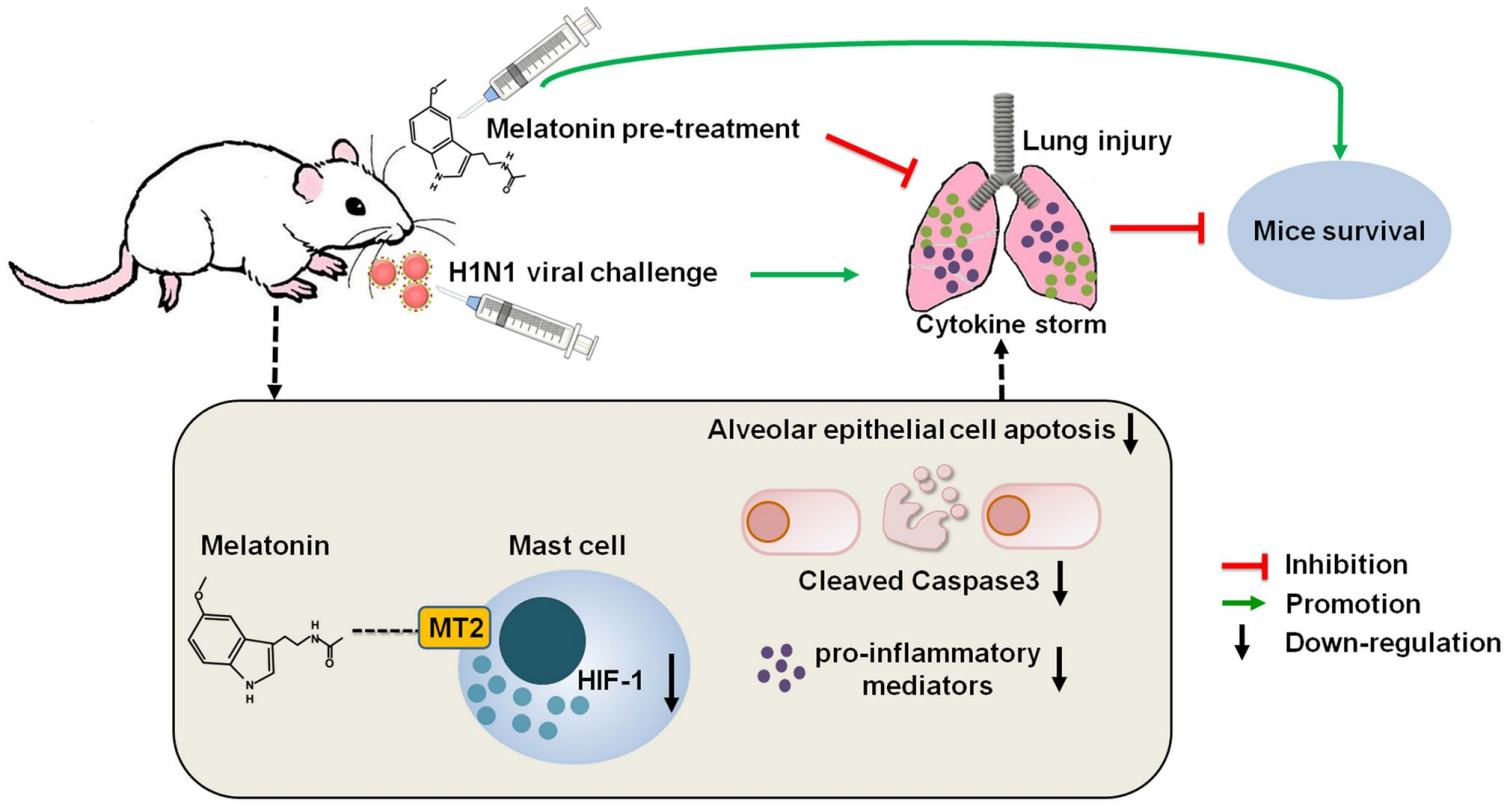
The summary illustration of potential mechanisms of melatonin in protecting mice against a H1N1 infection.

In summary, for the first time, we proved that melatonin has the capacity to suppress the activation of mast cells and the associated inflammatory response in an H1N1 infected cell line and in mice (Fig 7). The molecular mechanisms involve melatonin down-regulated of the expression of genes associated with HIF-1 pathway and inhibition of proinflammatory cytokines released by mast cells which are mediated by the MT2 receptor; these actions reduce the loss of alveolar epithelial cells due to apoptosis, therefore, inhibit lung injury. The findings provide a novel mechanism of melatonin to protect the H1N1-induced pulmonary injury. These results may facilitate the development of new drugs or strategies to fight H1N1 or other IAV virus infections.

## Materials and methods

### Ethics statement

All animal experiments were approved by the Animal Ethics Committee of China Agricultural University (approval number 201206078) and were performed in accordance to Regulations of Experimental Animals of Beijing Authority. Also, experimental protocols conformed to the guidelines of the Beijing Laboratory Animal Welfare and Ethics Committee and were approved by the Beijing Association for Science and Technology (approval number SYXK-2009-0423).

### Viruses and cell lines

The H1N1 (A/WSN/33) virus was provided by Dr. George F. Gao of the Institute of Microbiology, CAS, China, and the working stocks were generated in Madin-Darby canine kidney cells (MDCK) [60]. Virus titers were determined by standard plaque assay. The 50% lethal dose (LD50) in mice was determined as previously described [33]. The mouse mastocytoma cell line, P815, human lung adenocarcinoma cell line A549, and the MDCK were provided by the Cell Resource Center of Peking Union Medical College (Beijing, China) and cultured as described previously [61]. For *in vitro* virus inoculation, the virus was replicated in MDCK cells at 37°C for 48 h, and the viral supernatant was harvested, aliquoted, and stored at –80°C. The infectivity titer of the supernatant was measured in MDCK cells following serial dilution of the stock using a half-maximal tissue culture infectious dose (TCID50) assay and total titer was measured using a hemagglutination assay.

### Mice and treatments

#### Basal information collection

Twelve SPF female CD1 mouse aged 6 weeks were purchased from Beijing Vital River Laboratory Animal Technology Co., Ltd (Beijing, China). The mice were housed under controlled conditions with temperature (22–26°C) and 12-hour light: 12-hour dark cycle (with lights on at 06:00, off at 18:00), the light intensity at the cage level was around 750 lux. Animals were allowed access to food and water *ad libitum*. After adaptation for 1 week, the mice were anesthetized with isoflurane inhalation, and their vein blood, noses and lungs were collected at Zeitgeber Time of 18:00, 0:00. 6:00 and 12:00 hours. Tissues were ground with liquid nitrogen and suspended in PBS for preparing the homogenate, and then were centrifuged at 12000 × g for 10 min at 4°C to obtain the supernatant. The blood was centrifuged at 850 × g for 10 min to obtain the serum. The serum and supernatants were subjected to high performance liquid chromatography mass spectrometry analysis to confirm the levels of melatonin in CD1 mouse at different time points during one 24-hour period.

#### Survival study

Thirty SPF CD1 mice were divided into three groups (n = 10 for each group). After adaptation for 1 week, the mice were anesthetized with Zotile (1:7 in saline) by intramuscular injection, and then were intranasally infected with 120 TCID50/mouse H1N1 virus at 0:00 and 12:00 noon. The mice were observed daily for 14 days after viral challenge, and their survival rates were calculated to investigate their different susceptibilities.

#### Treatments

To investigate the potential protective effects of melatonin on the pathogenesis of lung injury induced by H1N1, we selected the 7-week wild type CD1 mouse (n = 7) and AANAT^-/-^ CD1 mouse (n = 7) which were generated from wild type CD1 mouse with the same genetic background by our group. The mice were anesthetized with Zotile (1:7 in saline) by intramuscular injection, and were intranasally infected with 120 TCID50/mouse H1N1 virus. Their survival rate, virus titers in lungs and tissue pathological changes were detected and analyzed.

The SPF female BALB/c mice were divided into eight groups (n = 15 for each group): the mock group, the melatonin 3, 10 and 30 mg/kg, intranasally (i.n.) treated groups, the H1N1 group, the H1N1 + melatonin 3 or 10 mg/kg, i.n. groups. Melatonin was given daily at Zeitgeber Time 3:00 hours for 3 days prior to H1N1 inoculation and the control infected group only received sterile saline. For melatonin delivery, isoflurane inhalation was selected to induce rapid and transient anesthesia. Then, the mice were intranasally infected with 120 TCID50/mouse H1N1 virus under the anesthesia of Zotile (1:7) intramuscular injection (i.m) which induced stable and relatively long anesthesia for virus inoculation. Thereafter, the survival rate and daily body weights of the mice were monitored and analyzed for 15 days. The mock group received only sterile saline without the infective agent.

The SPF female BALB/c mice were divided into four groups (n = 15 for each group): the mock group, the melatonin (10 mg/kg, i.n.) group, the H1N1 group, and the H1N1 + melatonin (10 mg/kg, i.n.) group. The mice from melatonin and H1N1 + melatonin group were given 25 μL melatonin solution daily for 3 days before H1N1 inoculation and the mock and H1N1 groups only received the same volume of sterile saline anesthetized with isoflurane inhalation. The mice were then, intranasally infected with 120 TCID50/mouse H1N1 virus anesthetized with Zotile (1:7). The mice were monitored and weighed daily after the viral challenge, and 5 mice in each group were sacrificed at days 3, 6 and 9 post-infections, respectively. The vein blood was collected and centrifuged to obtain the serum. The nose, trachea, and lung tissues were collected, half of which were fixed in 4% paraformaldehyde solution, and half frozen in liquid nitrogen and then were stored at –80°C. The details of the treatments were listed in S1 Table.

### High performance liquid chromatography (HLPC) mass spectrometry

The serum and supernatants were collected for melatonin measurement by HLPC mass spectrometry as previously described by Zhao et al [62].

### Histopathological changes

After 48 h fixation at room temperature, tissues were embedded in paraffin, and then cut into sections at 5 μm thickness. The samples were stained with hematoxylin and eosin (H&E) for histopathological evaluation by a veterinary pathologist and scored from 0 to 4 in a blind study. Descriptions of the scores were as follows: 0 = no microscopic lesions; 1 = extremely mild, characterized by slight hyperemia and hemorrhage; 2 = mild, characterized by hemorrhage, hyperemia, and desquamation of rare epithelial cells; 3 = moderate, characterized by hemorrhage, hyperemia, desquamation of many epithelial cells, and slight infiltration of inflammatory cells; 4 = severe, characterized by hemorrhage, hyperemia, desquamation of many epithelial cells, and greater infiltration of inflammatory cells.

### Immunohistochemistry

Paraffin sections were dewaxed, dehydrated, and immersed in distilled water, and then were incubated in 3% hydrogen peroxide solution for 15 min at room temperature. After washing with distilled water again, the sections were blocked with 1% bovine serum albumin for 20 min at room temperature. The sections were incubated with primary mouse anti-IAV nucleoprotein (NP) monoclonal antibody (1:1000, Abcam, Shanghai, China), and rabbit anti-mast cell tryptase monoclonal antibody (1:500, Abcam, Shanghai, China) antibodies overnight at 4°C. After rinsing with PBS, the sections were treated with goat anti-mouse and anti-rabbit immunoglobulin G (IgG) conjugated to horseradish peroxidase (Zhongshan Golden Bridge Biotechnology, Beijing, China) at room temperature for 1 h. After treating the sections with chromogen diaminobenzidine (Zhongshan Golden Bridge Biotechnology, Beijing, China) for 10 min at room temperature in the dark, the sections were counterstained with hematoxylin. The primary antibodies were replaced with PBS in the negative controls. The integral optical density (IOD) of H1N1 NP and tryptase proteins in the lung were identified in 10 high-power fields for each sample using Image-Pro plus 6.0, and the means were calculated. Sampling of the sections was unbiased; all the samples were coded and the examinations were performed by a single investigator.

### Toluidine blue staining

Mast cells were analyzed using an improved toluidine blue staining method [63]. Briefly, the paraffin sections were dewaxed, rehydrated, and immersed in 0.8% toluidine blue (Sigma, Shanghai, China) for 15 s. The sections were rinsed with distilled water, placed in 95% alcohol until the mast cells appeared deep reddish-purple; the sections were then dehydrated and mounted. The distribution of mast cells in the tissues was observed under a light microscope, and their numbers were counted and calculated by one inspector.

### Enzyme-linked immunosorbent assay (ELISA)

The concentrations of TNF-α, IL-1β, IL-6, histamine, and tryptase in the supernatants of mock- and virus-infected P815 cell cultures, the serum, homogenates of nose and lungs from control and virus-infected mice were determined using ELISA kits (eBioscience) according to the manufacturer’s instructions.

### Terminal-deoxynucleoitidyl transferase mediated nick end labeling (TUNEL)

The presence of apoptotic cells was determined using the In Situ Cell Death Detection Kit (Roche, Philadelphia, PA). Paraffin-embedded sections of lungs were dewaxed, rehydrated according to standard procedures, and immersed in a plastic jar containing 200 mL of citrate buffer (0.1 M, pH 6.0). The slides were subjected to microwave irradiation (750 W [high]) for 1 min, cooled rapidly by immediately adding 80 mL of double-distilled water, and then transferred into PBS. The slides were immersed for 30 min in Tris-HCl (0.1 M, pH 7.5, containing 3% BSA and 20% normal bovine serum) and then rinsed twice with PBS. The preceding steps were performed at 20–25°C. The TUNEL reaction mixture was added to the slides, which were then incubated at 37°C in a humidified atmosphere in the dark for 60 min. The slides were rinsed three times in PBS for 5 min each. Samples were analyzed in a drop of PBS using a fluorescence microscope with excitation and detection wavelengths of 450–500 nm and 515–565 nm (green), respectively. The TUNEL reaction mixture was replaced with label solution in the negative control, and the number of positive cells was counted in 10 high-power fields at ×200 magnification, and the means were calculated. Sampling of the sections was unbiased; all the samples were coded and the examinations were performed by a single investigator.

### Quantitative real-time PCR (RT-qPCR)

Total RNA was isolated from the lung samples using TRIzol reagent, according to the manufacturer’s protocol (Invitrogen, USA). The RNA was purified using a RNeasy mini kit (Qiagen, USA). The RNA quality and purity were determined using a NanoDrop ND-1000 spectrophotometer at 260/280 nm (NanoDrop Technologies, USA). Total RNA (0.5 μg) was transcribed into cDNA using the Fast Quant RT Kit (with gDNase) (Tiangen Biotech Co. LTD, Beijing, China) according to the manufacturer’s instructions. The expression levels of the genes with RT-qPCR using the SYBR Green Real-time PCR Master Mix (Tiangen Biotech Co. LTD, Beijing, China) were quantified. The primers were designed using the Primer Premier 5 software (PREMIER Biosoft, Palo Alto, CA) and were subsequently synthesized (Sangon Biotech Co. LTD, Beijing, China). The detected genes and primers were listed in S2 Table. The cycling parameters used for qPCR amplification were as follows: initial heat-denaturation at 95°C for 4 min; 40 cycles of 95°C for 30 s, 59–60°C for 30 s, and 72°C for 30 s; and a final extension at 72°C for 5 min. We performed a melting curve analysis to exclude genomic DNA contamination and to confirm primer specificities. Gene expression was normalized using the 2^-ΔΔCT^ method with the glyceraldehyde 3-phosphate dehydrogenase (*GAPDH*) gene used as an internal standard. Each biologic duplicate was controlled in 3 technical replicates.

### Cell culture and experiment design

To investigate the effect of melatonin on P815 and A549 cells, the adequate concentration of melatonin was initially determined using methyl thiazolyl tetrazolium (MTT) assay according to the manufacture’s protocol (Beijing Soleibao Technology Co., LTD, Beijing, China). The P815 and A549 cells were both divided into mock, melatonin (MT), MT + H1N1, and H1N1 groups (n = 3 for each group) when the action time of MT and H1N1 is 2 h and 1 h, respectively. The protective effect of melatonin on A549 cells was conducted by apoptotic analysis. The P815 cells exposed to H1N1 were collected to analyse its immune responses by detecting histamine, tryptase, and immune related genes expressions. The supernatant of P815 culture medium after exposed to MT and H1N1 were collected at 12 h, and then were added to the A549 cells to test whether they could protect A549 cells from H1N1 infection. All cells were treated with 10^−5^ mol/L melatonin for 2 h and then they were infected with viruses at 0.1 MOI (multiplicity of infection) for 1 h at 37°C. After washing, DMEM supplemented with 1% bovine serum albumin was added and cultured for the indicated periods at 37°C, 5% CO_2_ incubator.

The effects of MT2 receptor inhibitor 4P-PDOT (10^−7^ M, SML1189, Sigma) were also tested. The P815 cells were divided into mock, 4P-PDOT, MT, MT + H1N1, H1N1, MT + H1N1 + 4P-PDOT and H1N1 + 4P-PDOT groups (n = 3 for each group). Cells were cultured with melatonin and/or 4P-PDOT for 2 h at 37°C, 5% CO_2_ and the cells were collected and stored at −80°C for future assays.

### RNA sequencing (RNA-seq) and data analysis

To analyse differentially expressed genes (DEGs) in the P815 cells after exposure to melatonin, the cells from mock, MT + H1N1, and H1N1 group (n = 3 for each group) were collected for transcriptome analysis. Total RNA was isolated using the TRIZOL reagent (Invitrogen, USA) according to the manufacturer’s instructions. We visualized RNA degradation and contamination on 1% agarose gels; RNA purity was checked using a NanoPhotometer spectrophotometer (IMPLEN), and concentrations were determined using the Qubit^®^ RNA Assay Kit in Qubit 2.0 Fluorometer (Life Technologies, CA, USA). We assessed RNA integrity using the RNA Nano 6000 Assay Kit of the Bioanalyzer 2100 system (Agilent Technologies), and a total of 3μg of RNA from each sample was used as the input material for RNA sample preparations. The ribosomal RNA was removed using an Epicentre Ribo-zero rRNA Removal Kit (Epicentre), and the mRNA sequencing libraries were constructed using an NEBNext Ultra Directional RNA Library Prep Kit for Illumina (NEB), according to the manufacturer’s recommendations. We then sequenced the mRNA libraries on an Illumina Hiseq 2000 platform and generated 100-bp paired-end reads.

The transcriptome sequencing and analysis were conducted by BGISEQ (BGI genomics) (Wuhan, China). Raw data (raw reads) were processed using Trimmomatic. The reads containing Ploy-N and the low-quality reads were removed to obtain the clean reads. Then the clean reads were mapped to the *Mus musculus* genome GCF_000001635.26_GRCm38.p6 genome sequence from NCBI using Bowtie2. Clean reads were mapped to reference transcripts, and then calculate the gene expression level for each sample with RSEM. DEGs were identified using the DESeq2. R package functions estimate SizeFactors and nbinomTest. Pvalue < 0.05 and log2-transformed fold changes > 2 were set as the threshold for significantly differential expression. Hierarchical cluster analysis of DEGs was performed to explore gene expression pattern. Gene Ontology (GO) enrichment and Kyoto Encyclopedia of Genes and Genomes (KEGG) pathway enrichment analysis of DEGs were respectively performed using R based on the hypergeometric distribution.

### Immunofluorescence

The replications of H1N1 virus in the cells were determined after detecting expression of IAV NP by immunofluorescence staining. Cultured cells were twice rinsed with PBS (0.01 M, pH 7.2) and then were fixed with 4% paraformaldehyde solution for 15 min at room temperature. After rinsing with PBS, the cells were permeabilized with 0.5% Triton X-100 for 15 min, and then rinsed and blocked with 3% BSA for 20 min at room temperature. The cells were incubated with mouse anti-IAV NP monoclonal antibody (1:1000, Abcam, Shanghai, China) overnight at 4°C. After rinsing three times with PBS, the cells were incubated with a FITC-conjugated goat anti-mouse secondary antibody (1:500, Abcam, Shanghai, China) for 1h at room temperature. To visualize the nuclei, cells were stained with 3 μg/ml 40, 60—diamidine-2-phenylindole (DAPI) (Sigma-Aldrich, Shanghai, China) for 5 min at room temperature and then they were examined under an inverted fluorescence microscope (Leica Microsystems, Wetzlar, Germany).

### Flow cytometric analysis of apoptosis

The cell apoptotic responses were examined at 12 h after infection using an Annexin V-FITC Apoptosis Detection Kit (eBioscience, San Diego, CA, U.S.) according to the manufacturer’s instructions. Flow cytometric analysis was performed on a BD FACSCalibur using Cell Questpro software (BD Biosciences). Taxol (Sigma-Aldrich, Beijing, China) at concentration of 25 nM served as a positive inducer of apoptosis.

### Western blot

Cells were harvested at 12 h after infection, washed with PBS, and lysed with RIPA lysis buffer containing protease inhibitor cocktail (Beyotime Institute of Biotechnology, Beijing, China). Protein concentrations were determined using a BCA protein assay kit (Beyotime Institute of Biotechnology). Equal amounts of protein were separated on 12% SDS-PAGE gel and transferred to a polyvinylidene difluoride (PVDF) membrane (Millipore, Beijing, China). The membranes were blocked using 5% non-fat dry milk (BD Biosciences) at room temperature for 2 h, washed, and probed using the specified antibodies. The rabbit anti-HIF-1α antibody and mouse anti-p300 antibody were obtained from Abcam Company and Santa Cruz Biotechnology, respectively. The rabbit anti-β-actin antibody, anti-caspase 3 antibody and anti-α-tubulin antibody as well as the corresponding horseradish-peroxide-conjugated secondary antibodies were obtained from Cell Signaling Technologies. Protein bands were visualized using Western Lightning Plus-ECL (Perkin Elmer, MA, U.S.). Tubulin served as a loading control.

### Statistical analysis

The data were expressed as mean ± SEM. The two-way analysis of variance (ANOVA) was used for the normality analysis and followed by Bonferroni statistical tests to compare the difference between groups with GraphPad Prism 8.0. P < 0.05 was considered as statistical significance.

## Supporting information

S1 Fig Effects of H1N1 virus infection on the histology of nose and trachea in WT and AANAT^-/-^ CD1 mice. The histology was analyzed at day 9 of post-infection by H&E staining and scored by an examiner blinded to the study. Black arrows indicate lymphocytic infiltration. Values were means ± SEM. (*P < 0.05, **P < 0.01) vs its respective group, determined by two-way ANOVA followed by Bonferroni statistical tests.
(PDF)

S2 Fig Effects of different doses of melatonin on the body weight of BALB/c mice infected by H1N1 virus. MT: melatonin. n = 15.
(PDF)

S3 Fig Effects of melatonin treatment on the histology of nose, trachea and lung of BALB/c mice infected by H1N1 virus. Tissues were collected at day 3, 6 and 9 of post-infection with H&E staining and scored by an examiner blinded to the study. Black arrows indicate lymphocytic infiltration. Values were means ± SEM. (*P < 0.05, **P < 0.01) vs its respective group, determined by two-way ANOVA followed by Bonferroni statistical tests.
(PDF)

S4 Fig Effects of melatonin treatment on the A549 cells infected by H1N1 virus. (A) Cell viability after melatonin (10^−5^ mol/L) treatment and/or H1N1 virus inoculation. (B) The replication of H1N1 virus at 12 h after post-infection with immunofluorescence staining. Green indicates NP; Blue indicates DAPI for nuclei. (C) Apoptosis with flow cytometric analysis. MT: melatonin.
(PDF)

S5 Fig Effects of melatonin treatment on the mast cells in the trachea of BALB/c mice. Numbers of mast cells in the trachea of mice after melatonin (10 mg/kg) treatment and H1N1 infection was measured at 3 d, 6 d and 9 d post-infection with toluidine blue staining, respectively. MT: melatonin. Values were means ± SEM. (*P < 0.05) vs its respective group, determined by two-way ANOVA followed by Bonferroni statistical tests.
(PDF)

S6 Fig Effects of melatonin treatment on P815 cells (mast cell) infected by H1N1 virus. (A) Dose response test of melatonin with MTT assay. (B) Cell viability after melatonin (10^−5^ mol/L) treatment and/or H1N1 virus inoculation was measured at 12 h after post-infection.
(PDF)

S1 Table The details of animal study designs. (DOCX)

S2 Table The primers were designed and used to detect the targeted genes by qPCR. (DOCX)
