# Supplementary figures and images for: Melatonin alleviates lung injury in H1N1-infected mice by mast cell inactivation and cytokine storm suppression
Source: PLoS Pathog. 2023 May 18;19(5):e1011406. doi: 10.1371/journal.ppat.1011406 (PMC10249807; doi:10.1371/journal.ppat.1011406)

**ANNAT<sup>-/-</sup>**

**Nose**

**Trachea**

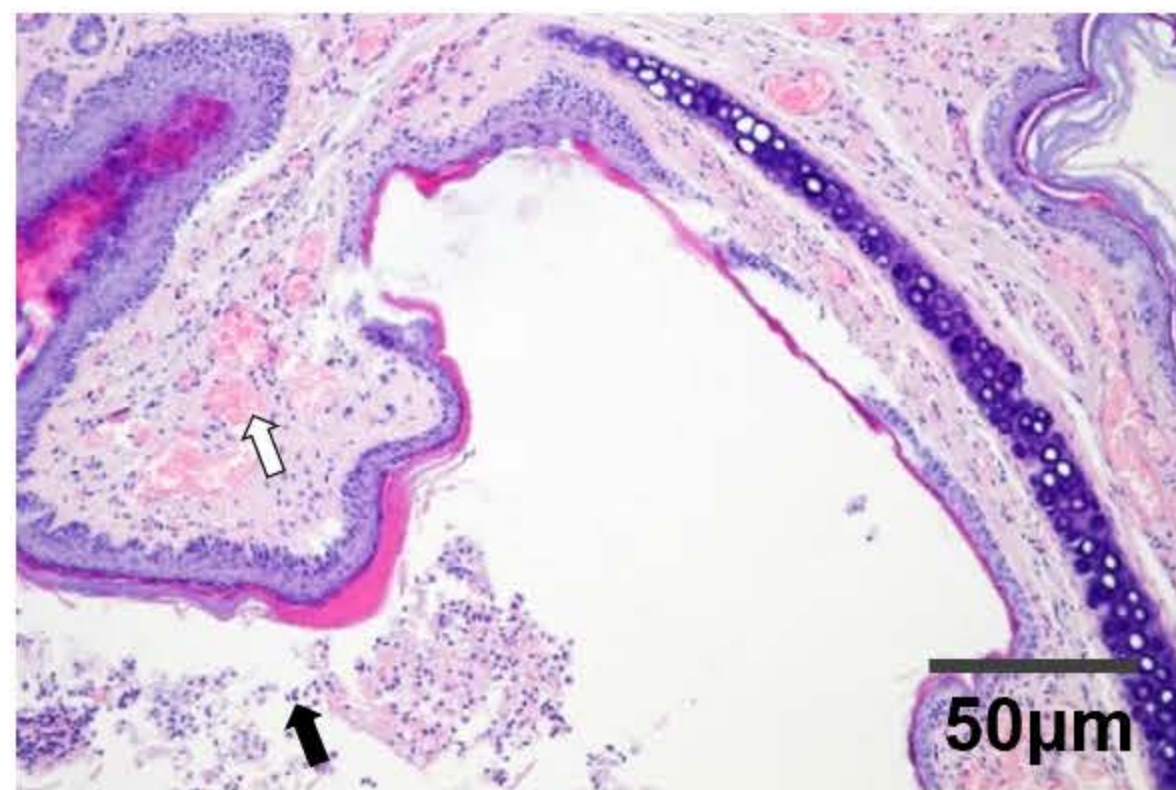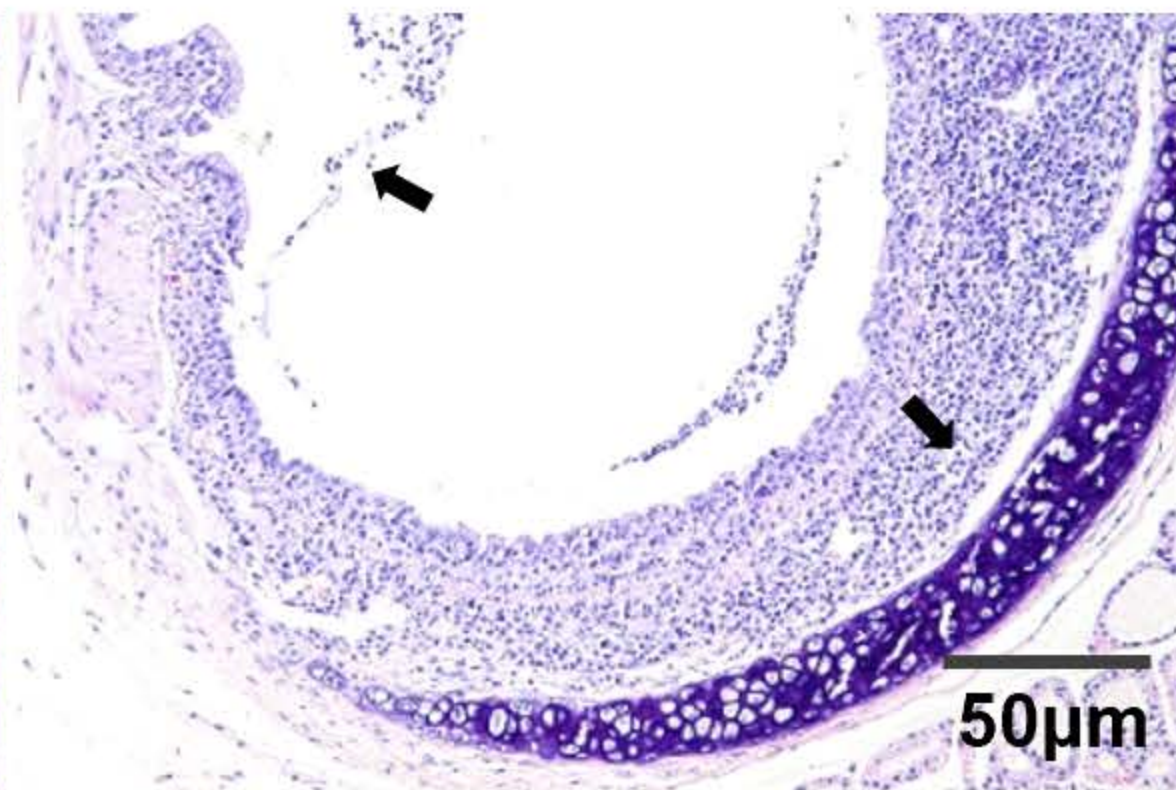

**WT**

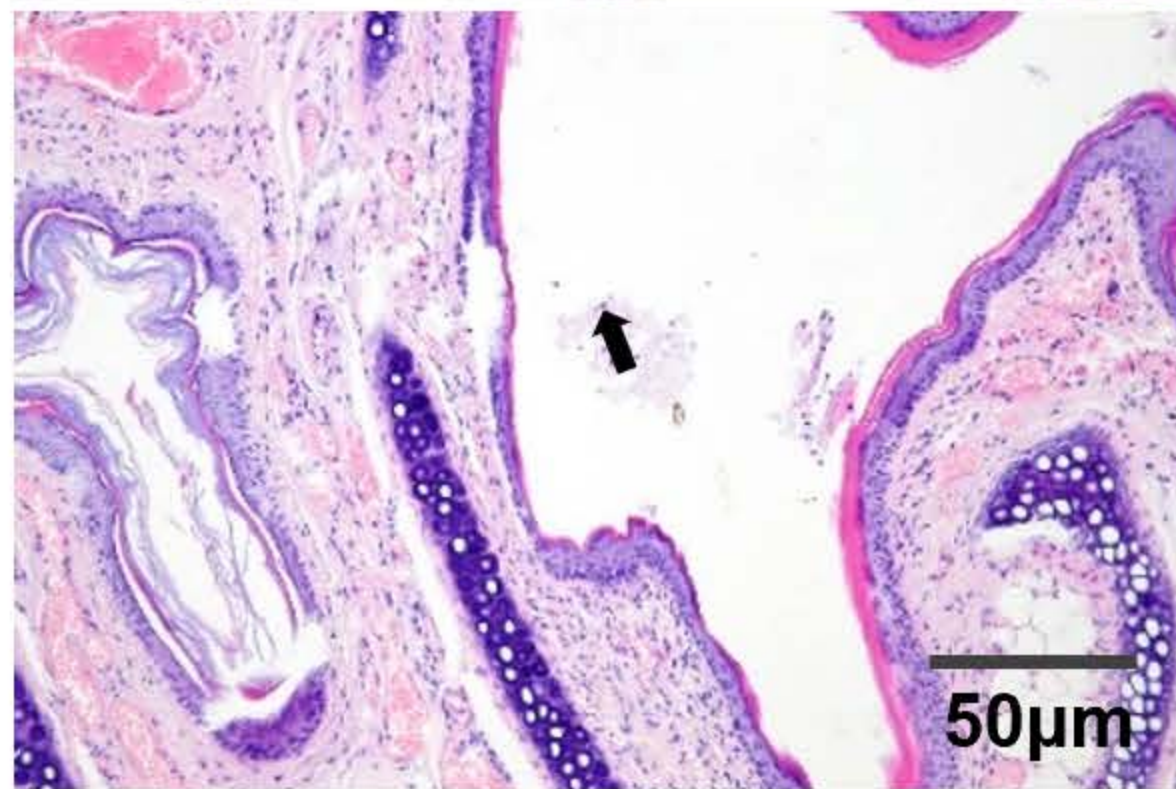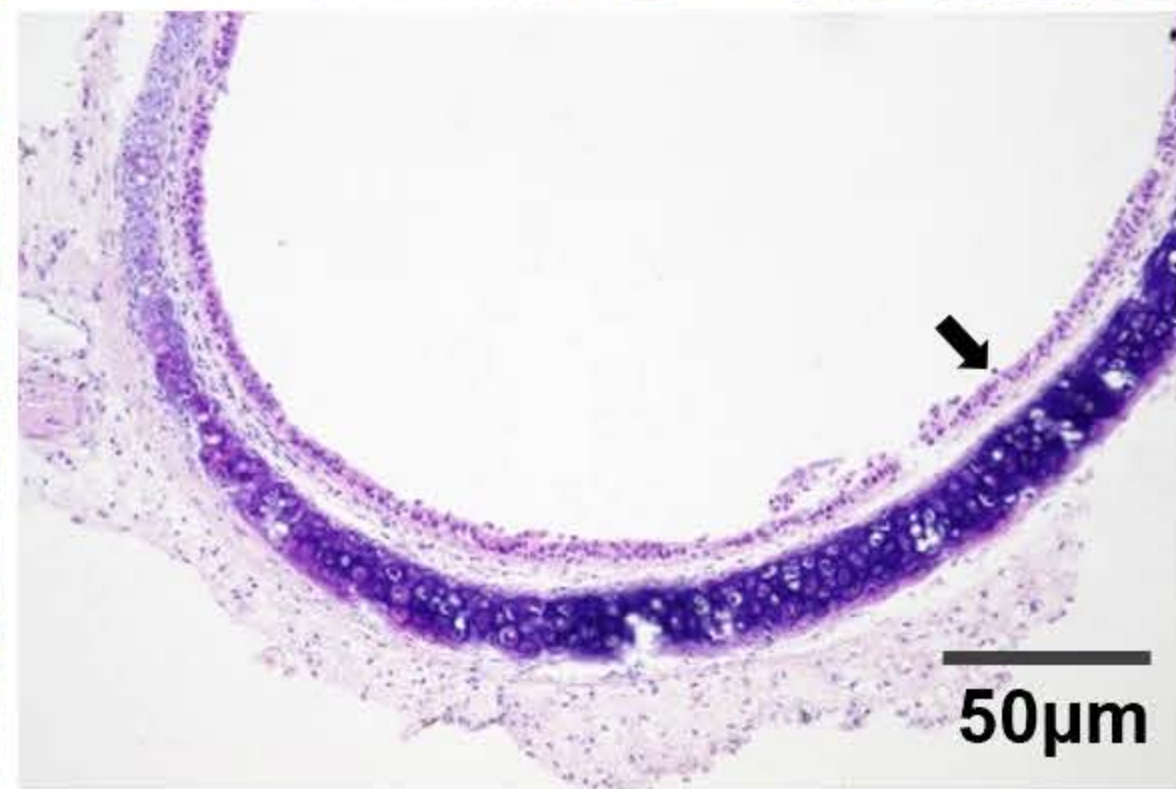

**Scores of microscopic lesions**

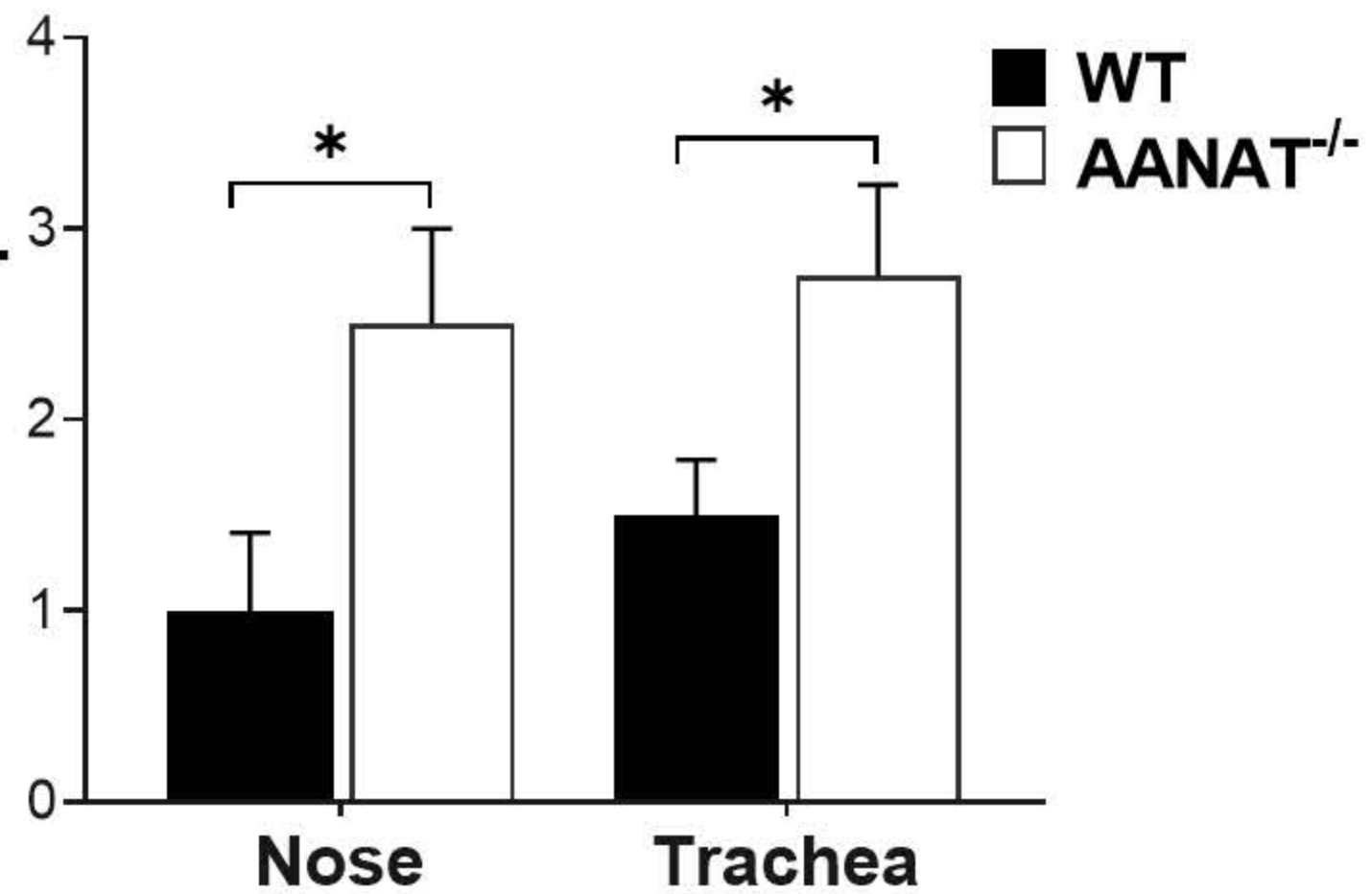

Supplement: S1 Fig — The histology was analyzed at day 9 of post-infection by H&E staining and scored by an examiner blinded to the study. Black arrows indicate lymphocytic infiltration. Values were means ± SEM. (*P < 0.05, **P < 0.01) vs its respective group, determined by two-way ANOVA followed by Bonferroni statistical tests. (PDF) [file ppat.1011406.s001.pdf]

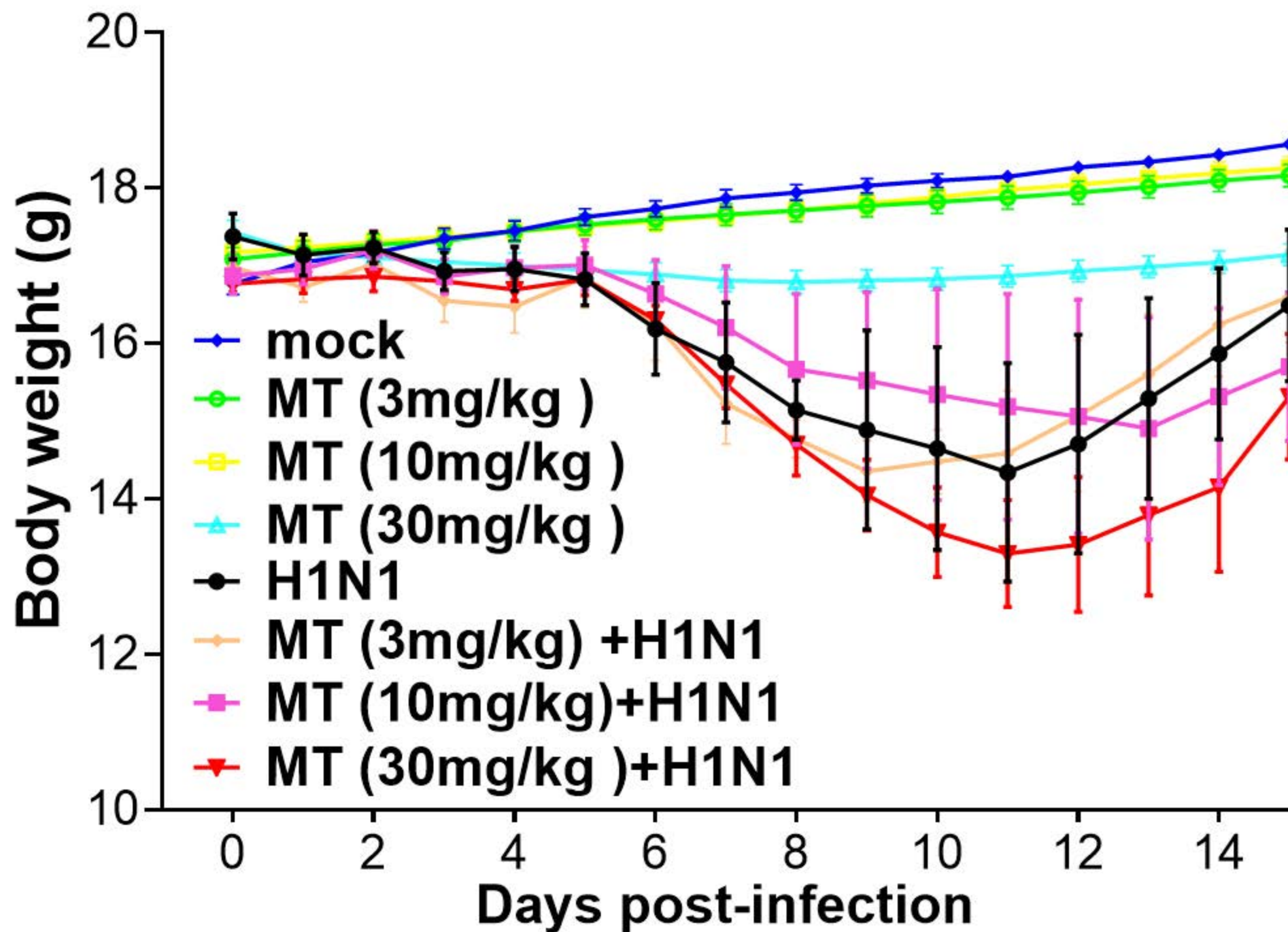

Supplement: S2 Fig — MT: melatonin. n = 15. (PDF) [file ppat.1011406.s002.pdf]

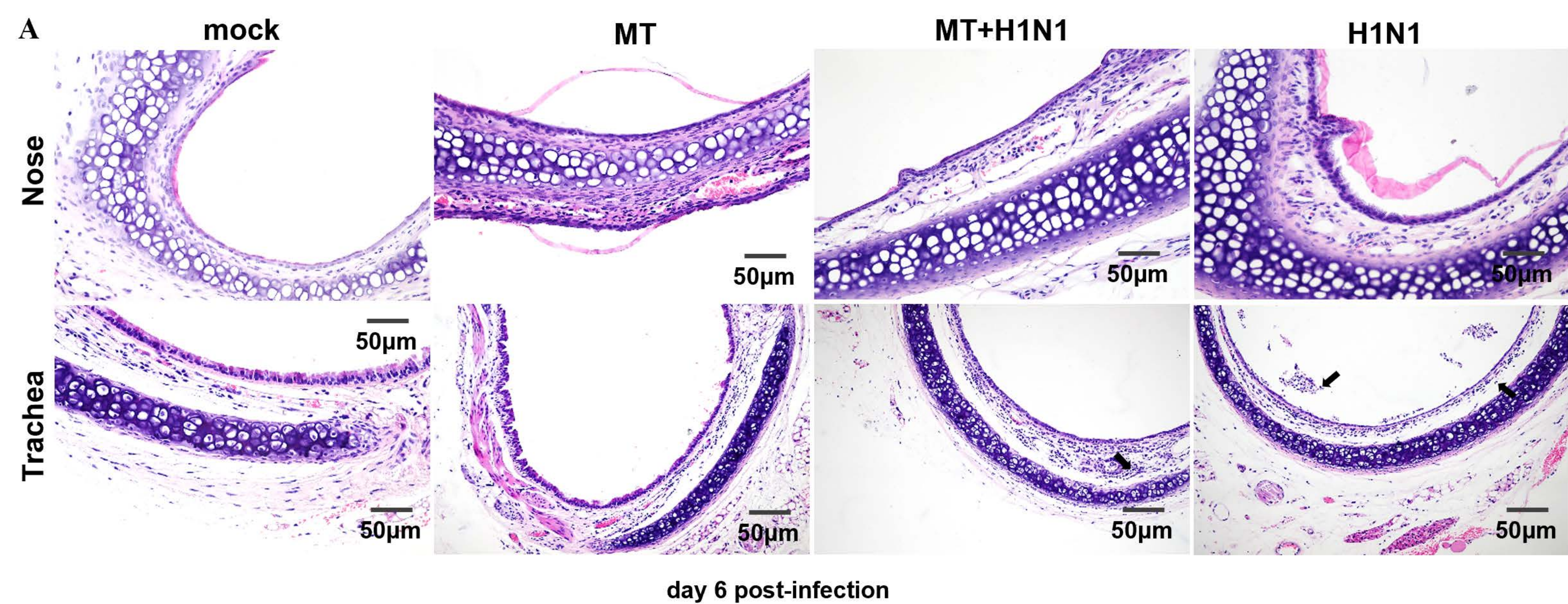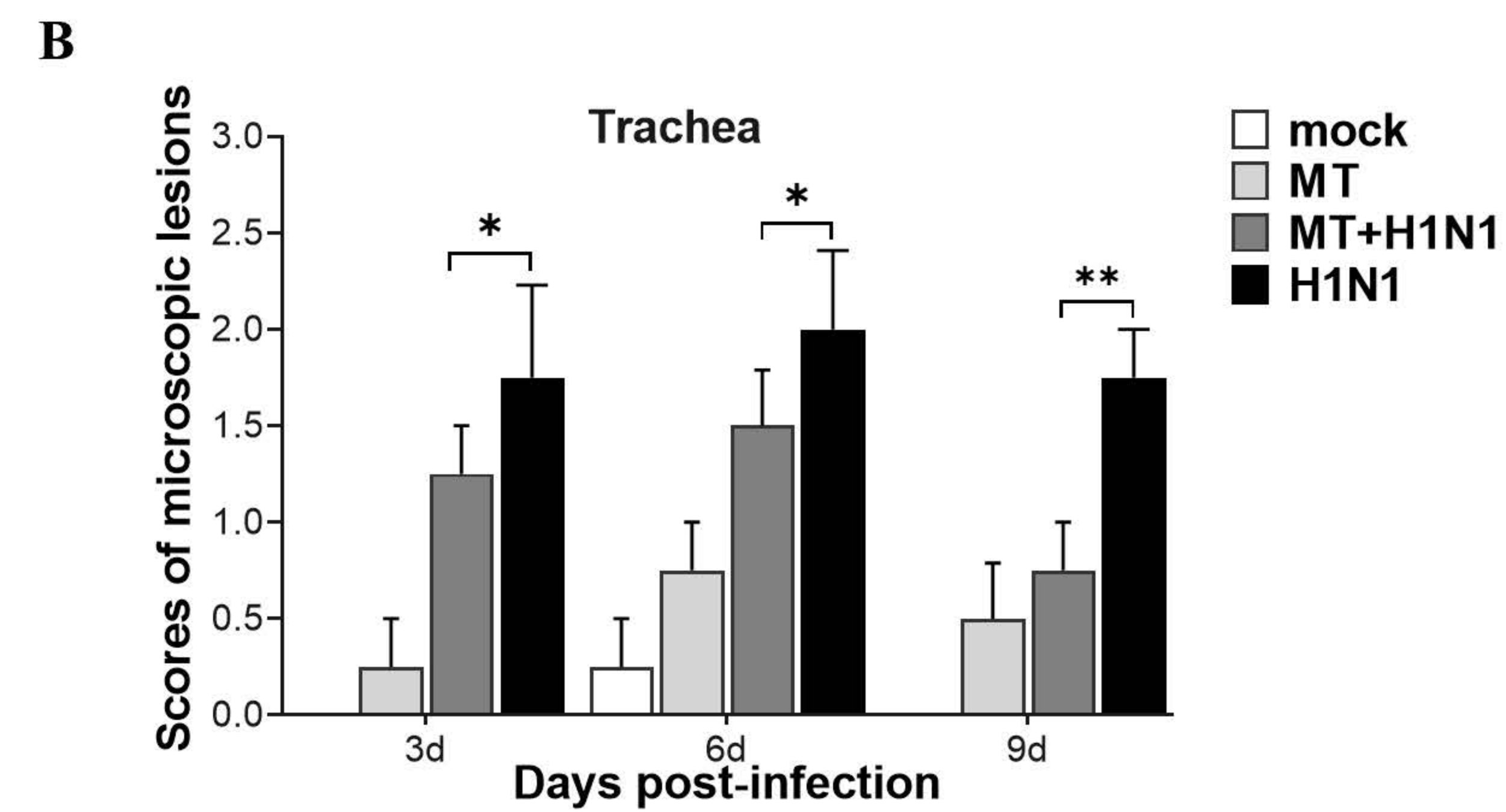

Supplement: S3 Fig — Tissues were collected at day 3, 6 and 9 of post-infection with H&E staining and scored by an examiner blinded to the study. Black arrows indicate lymphocytic infiltration. Values were means ± SEM. (*P < 0.05, **P < 0.01) vs its respective group, determined by two-way ANOVA followed by Bonferroni statistical tests. (PDF) [file ppat.1011406.s003.pdf]

**A**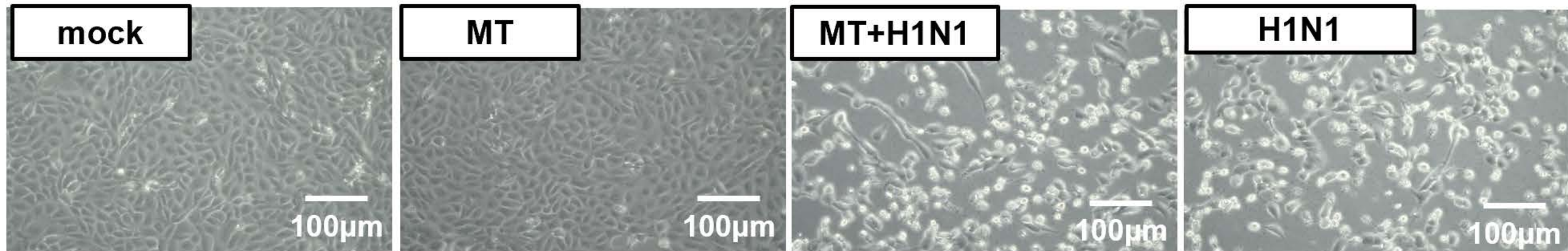**B**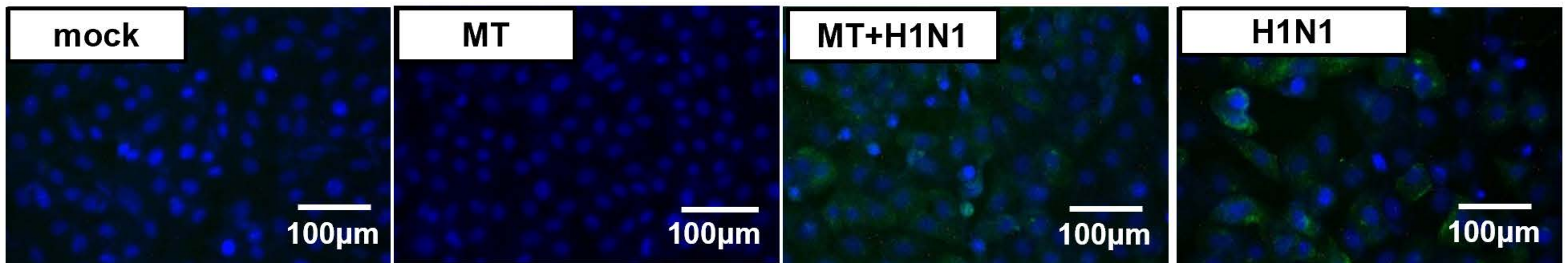**C**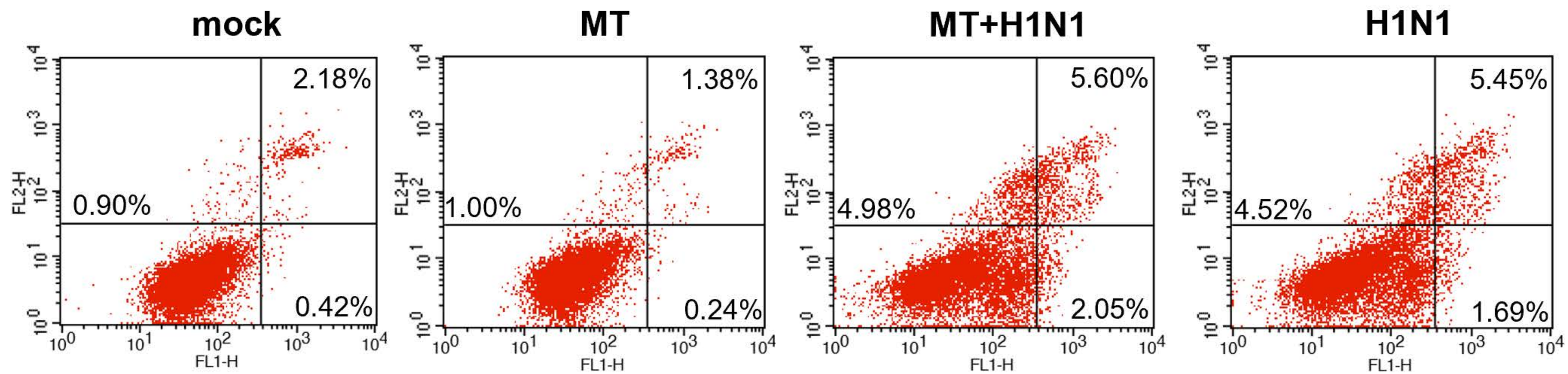

Supplement: S4 Fig — (A) Cell viability after melatonin (10−5 mol/L) treatment and/or H1N1 virus inoculation. (B) The replication of H1N1 virus at 12 h after post-infection with immunofluorescence staining. Green indicates NP; Blue indicates DAPI for nuclei. (C) Apoptosis with flow cytometric analysis. MT: melatonin. (PDF) [file ppat.1011406.s004.pdf]

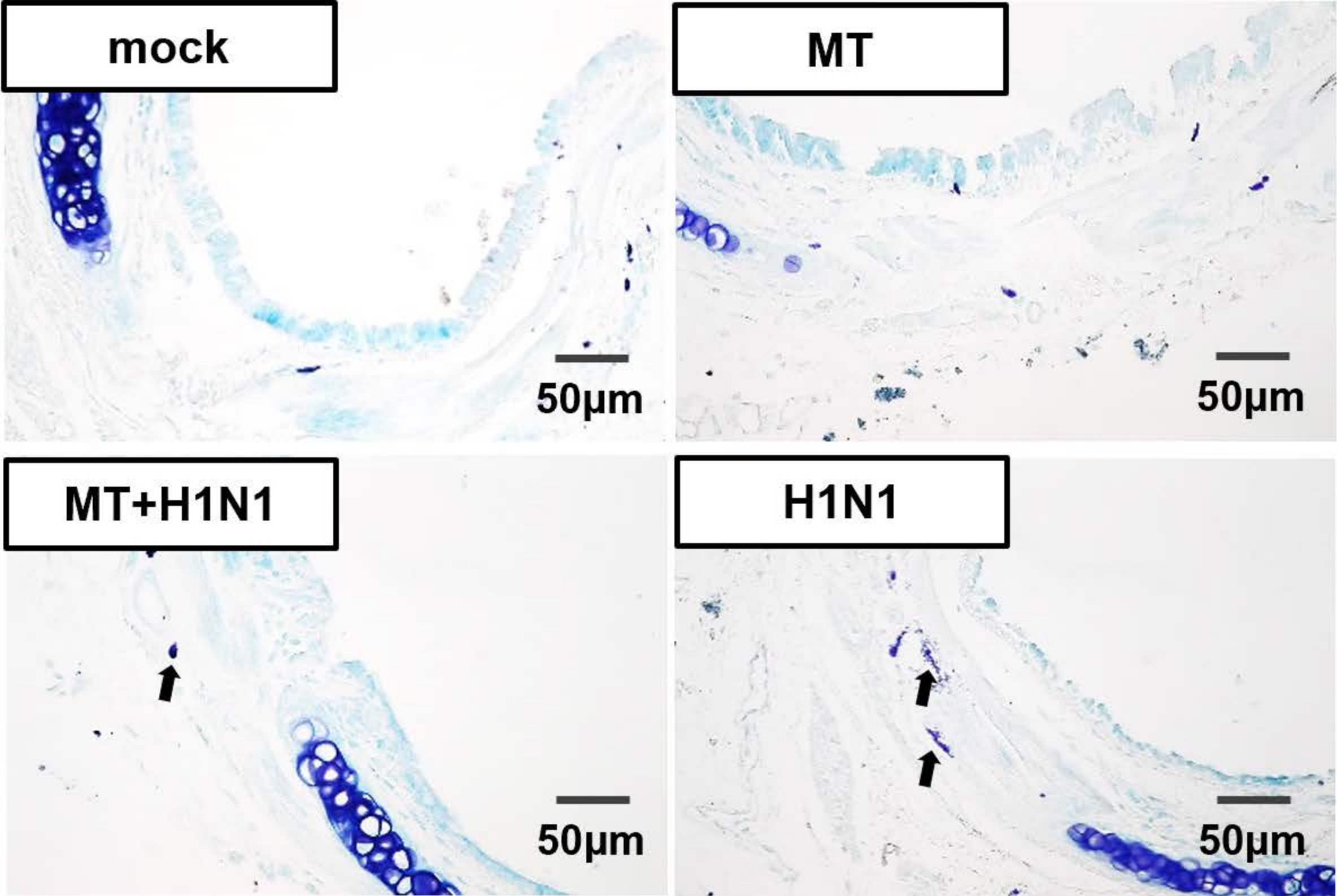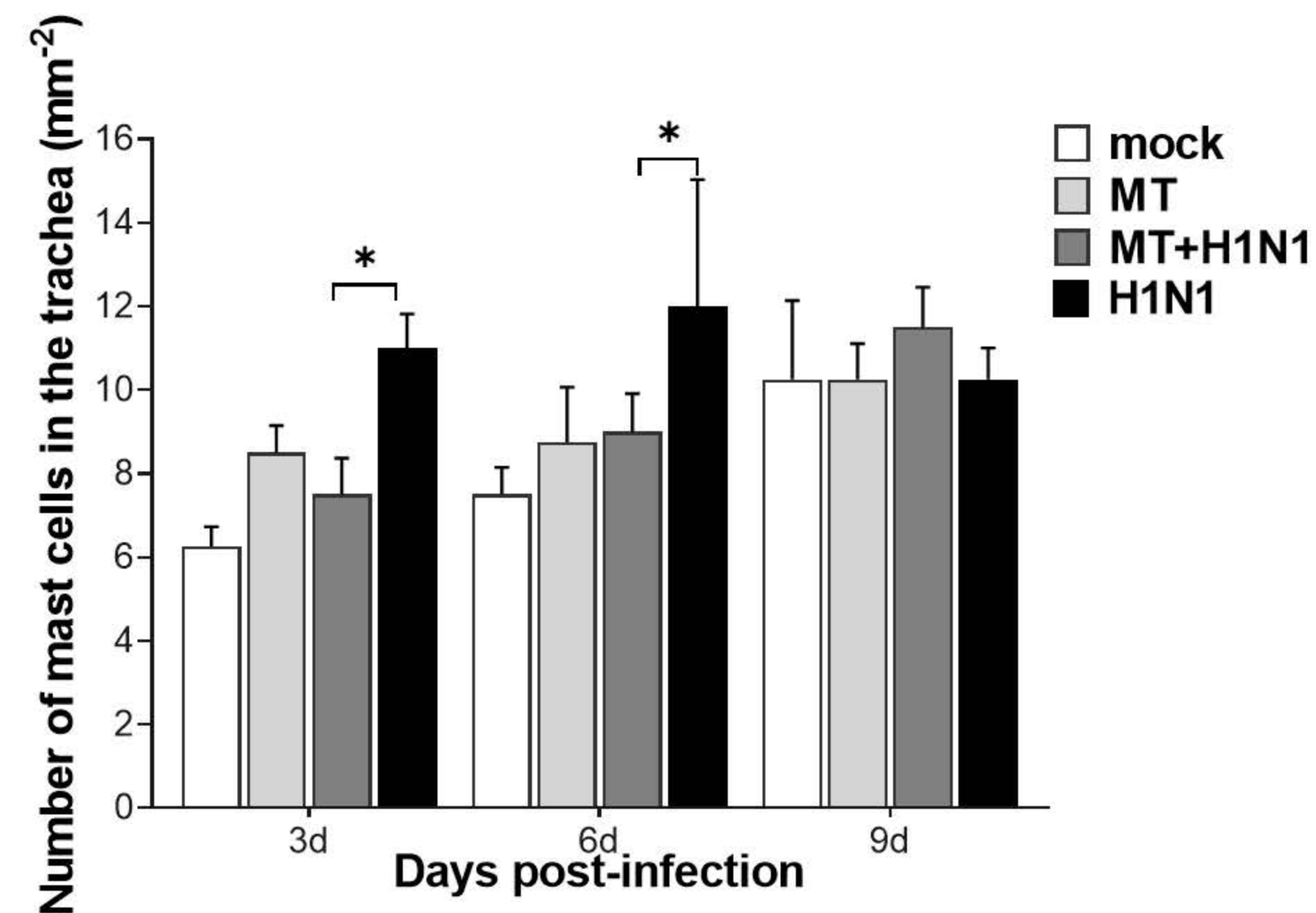

Supplement: S5 Fig — Numbers of mast cells in the trachea of mice after melatonin (10 mg/kg) treatment and H1N1 infection was measured at 3 d, 6 d and 9 d post-infection with toluidine blue staining, respectively. MT: melatonin. Values were means ± SEM. (*P < 0.05) vs its respective group, determined by two-way ANOVA followed by Bonferroni statistical tests. (PDF) [file ppat.1011406.s005.pdf]

**A**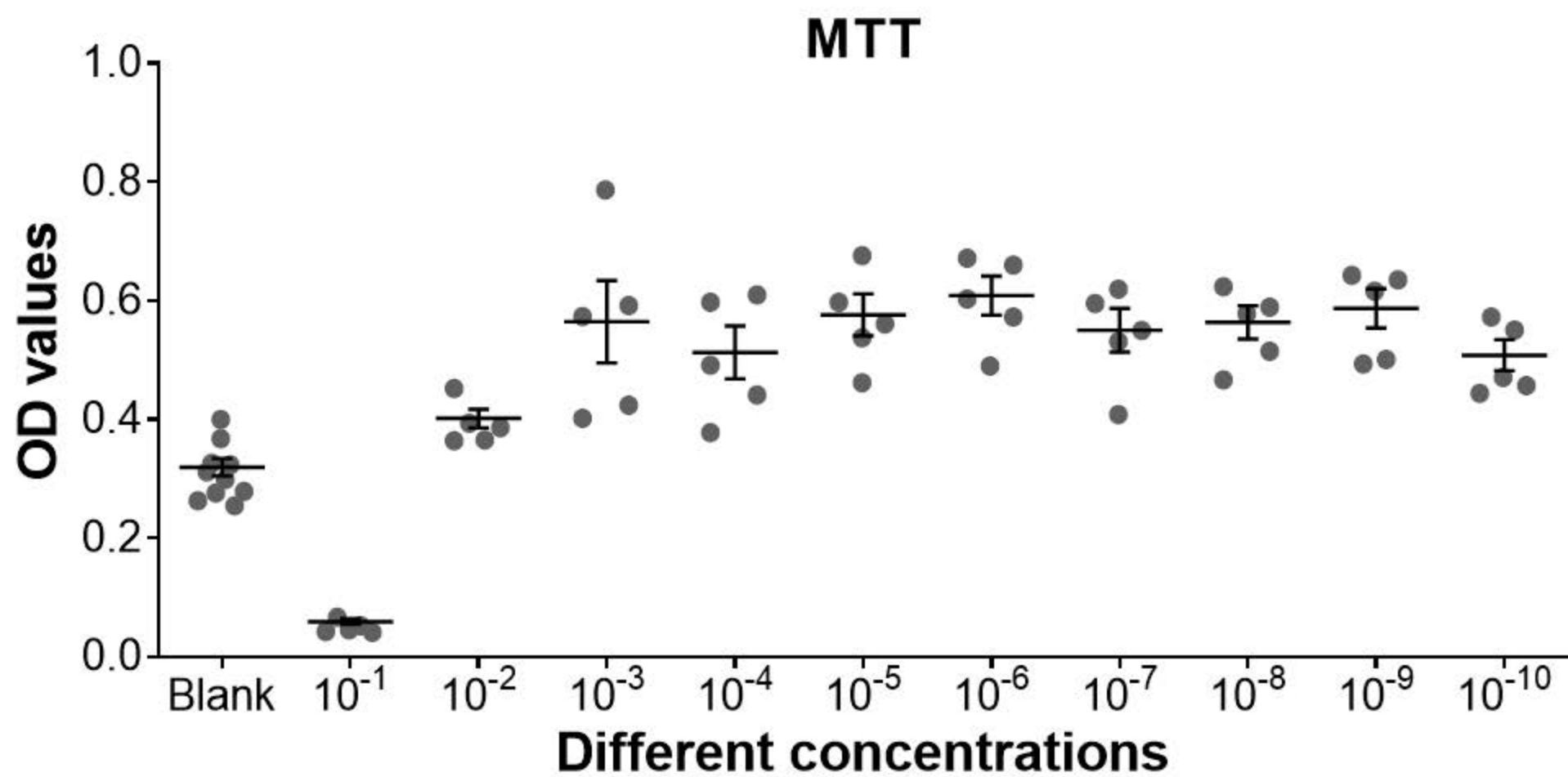**B**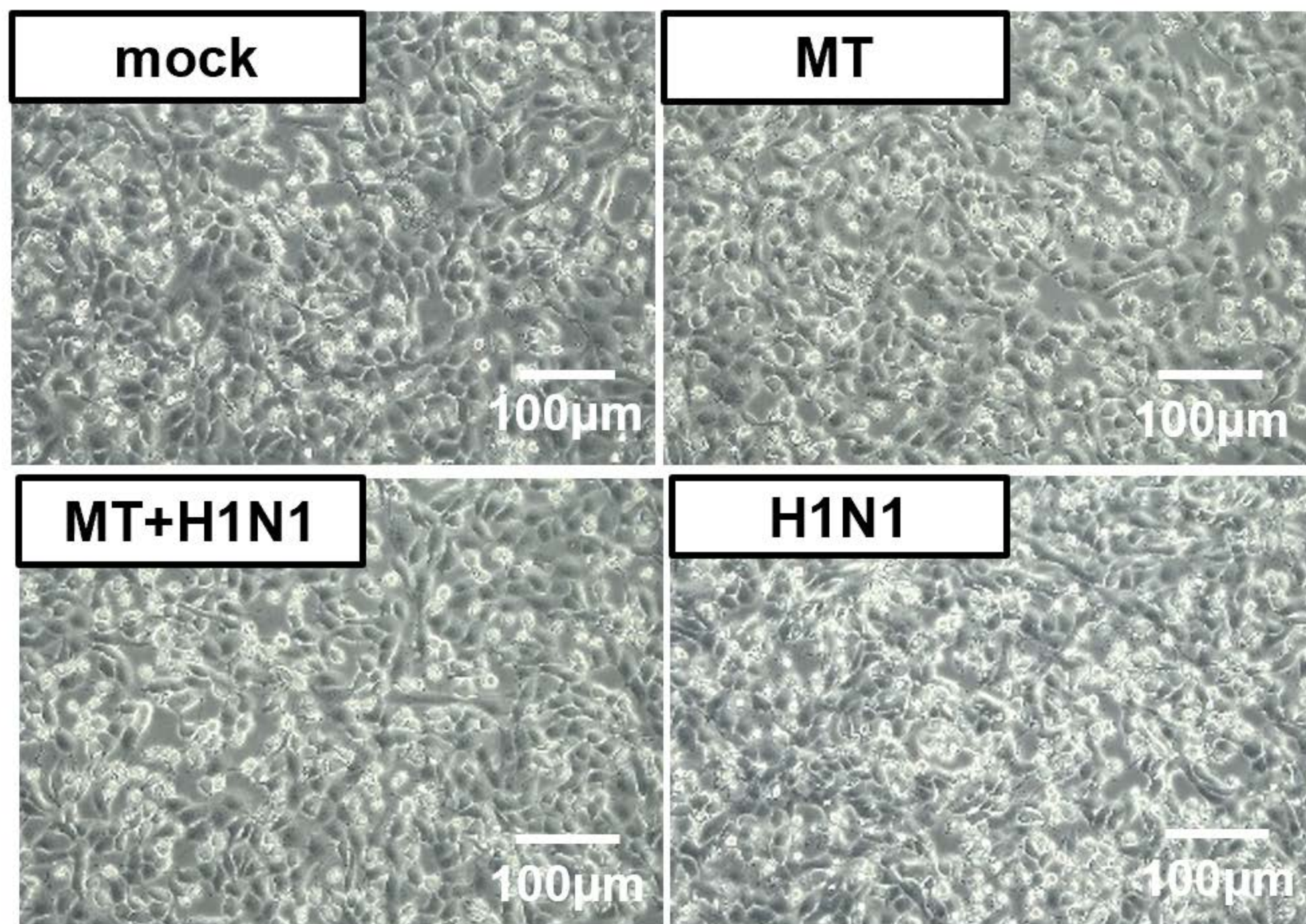

Supplement: S6 Fig — (A) Dose response test of melatonin with MTT assay. (B) Cell viability after melatonin (10−5 mol/L) treatment and/or H1N1 virus inoculation was measured at 12 h after post-infection. (PDF) [file ppat.1011406.s006.pdf]
