# Supplementary material for: Melatonin alleviates lung injury in H1N1-infected mice by mast cell inactivation and cytokine storm suppression
Source: PLoS Pathog. 2023 May 18;19(5):e1011406. doi: 10.1371/journal.ppat.1011406 (PMC10249807; doi:10.1371/journal.ppat.1011406)
Supplement: S1 Table — (DOCX) [file ppat.1011406.s007.docx]

**S1 Table. The details of animal study designs**

| Experiment | | Mice strain | Groups | Isoflurane  (inhalation) | Melatonin  (intranasal injection) | Zotile®  (intramuscular injection) | H1N1  (intranasal injection) |
| --- | --- | --- | --- | --- | --- | --- | --- |
| Basal information collection | | CD1  (n= 3 per group) | 0:00 AM  6:00 AM  12:00 AM  6:00 PM | +  +  +  + | -  -  -  - | -  -  -  - | -  -  -  - |
| Survival study | | CD1  (n= 10 per group) | mock  0:00 AM  12:00 AM | -  -  - | -  -  - | -  +  + | -  + (infect at 0:00)  + (infect at 12:00) |
| Treatments | i | CD1  (n= 7 per group) | WT  AANAT^-/-^ | -  - | -  - | +  + | +  + |
|  | ii | BALB/c  (n= 15 per group) | Mock  MT (3mg/kg)  MT (10mg/kg)  MT (30mg/kg)  H1N1  MT (3mg/kg) + H1N1  MT (10mg/kg) + H1N1  MT (30mg/kg) + H1N1 | -  +  +  +  -  +  +  + | -  + (3mg/kg)  + (10mg/kg)  + (30mg/kg)  -  + (3mg/kg)  + (10mg/kg)  + (30mg/kg) | -  -  -  -  +  +  +  + | -  -  -  -  +  +  +  + |
|  | iii | BALB/c  (n= 15 per group) | mock  MT (10mg/kg)  H1N1  MT (10mg/kg) + H1N1 | -  +  -  + | -  + (10mg/kg)  -  + (10mg/kg) | -  -  +  + | -  -  +  + |
