# Supplementary material for: Melatonin alleviates lung injury in H1N1-infected mice by mast cell inactivation and cytokine storm suppression
Source: PLoS Pathog. 2023 May 18;19(5):e1011406. doi: 10.1371/journal.ppat.1011406 (PMC10249807; doi:10.1371/journal.ppat.1011406)
Supplement: S2 Table — (DOCX) [file ppat.1011406.s008.docx]

**S2 Table. The primers were designed and used to detect the targeted genes by qPCR**

| Genes | Primer sequence (5’-3’) |
| --- | --- |
| NS1 | F-GCAATTGGAATCCTCATCGG |
|  | R-CAACTCGTTTCGCCATGTAGC |
| MT1-M | F-CCATTTCATCGTGCCTATG |
|  | R-GTAACTAGCCACGAACAGC |
| MT2-M | F-TACATCAGCCTCGTCTGGCTCC |
|  | R-TTCCTCGTAGCCTTGGCCTTCC |
| GAPDH-M | F-TGTGAACGGATTTGGCCGTA |
|  | R-ACTGTGCCGTTGAATTTGCC |
| GAPDH-H | F-TTCTTTTGCGTCGCCAGCC |
|  | R-GCCATGGGTGGAATCATATTGG |
